# Supplementary material for: Engineering natural microbiomes toward enhanced bioremediation by microbiome modeling
Source: Nat Commun. 2024 Jun 1;15:4694. doi: 10.1038/s41467-024-49098-z (PMC11144243; doi:10.1038/s41467-024-49098-z)
Supplement: Supplementary file 1 — Supplementary Information [file 41467_2024_49098_MOESM1_ESM.pdf]

Engineering natural microbiomes toward enhanced bioremediation by microbiome modeling

Zhepu Ruan[1,2#], Kai Chen[1#], Weimiao Cao[1], Lei Meng[1], Bingang Yang[1], Mengjun Xu[1], Youwen Xing[1], Pengfa Li[1], Shiri Freilich[3], Chen Chen[1], Yanzheng Gao[4*], Jiandong Jiang[1*], Xihui Xu[1*]

1 Department of Microbiology, College of Life Sciences, Nanjing Agricultural University, Key Laboratory of Agricultural and Environmental Microbiology, Ministry of Agriculture and Rural Affairs, Nanjing 210095, China

2 Guangdong Laboratory for Lingnan Modern Agriculture, Guangdong Provincial Key Laboratory of Agricultural & Rural Pollution Abatement and Environmental Safety, College of Natural Resources and Environment, South China Agricultural University, Guangzhou 510642, China

3 Newe Ya'ar Research Center, Agricultural Research Organization, P.O. Box 1021, Ramat Yishay 30095, Israel

4 College of Resources and Environmental Sciences, Nanjing Agricultural University, Nanjing 210095, China

# These authors contributed equally to this work.

* Corresponding authors:

Email address: gaoyanzheng@njau.edu.cn (Y. Gao)

Email address: jiang_jjd@njau.edu.cn (J. Jiang)

Email address: xuxihui@njau.edu.cn (X. Xu)

Supplementary Information

Supplementary Figures

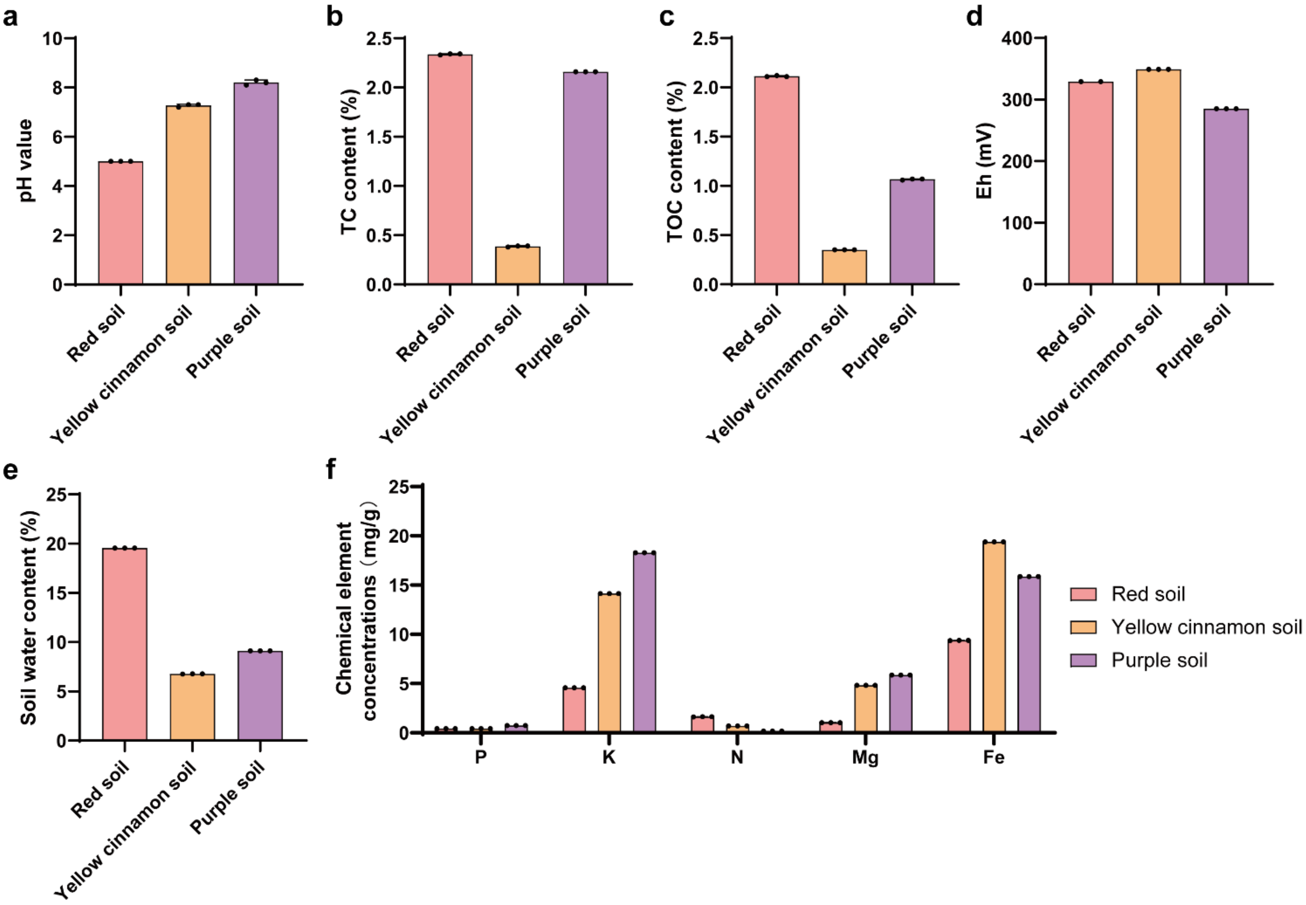

Supplementary Fig. 1. Basic chemical properties of three soils used in this study. **a** pH value. **b** Total carbon (TC) content. **c** Total organic carbon (TOC) content. **d** Soil redox potential (Eh). **e** Soil water content. **f** Chemical element concentrations. The data are presented as mean values ± SD (n = 3 biological independent replicates). Source data are provided as a Source Data file.

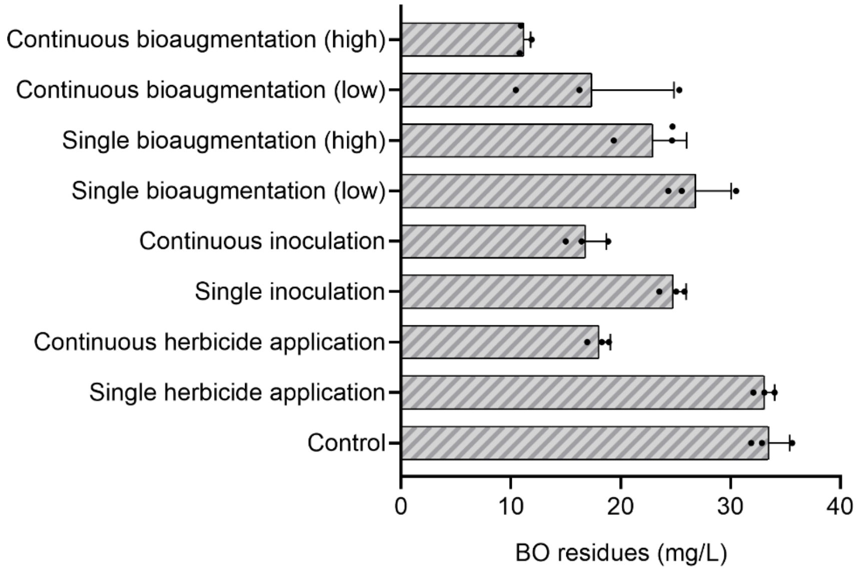

Supplementary Fig. 2. Degradation ability of bromoxynil octanoate (BO) at day 30 by different treated microbiomes. Control, only regular water spray; Single and continuous herbicide application: adding 5 mg/kg of BO once (Single) or every three days for 10 times (continuous); Single and continuous inoculation: inoculating two strains X-1 and 7D-2 at a ratio of 1:1, with a final concentration of approximately 2×10^6 CFU/g soil for each strain, once (Single) or every three days for 10 times (continuous); Single bioaugmentation (low and high): inoculating two strains X-1 and 7D-2 at a ratio of 1:1, with a final concentration of approximately 2×10^6 (low) or 2×10^8 (high) CFU/g soil for each strain, and adding 5 mg/kg of BO only once; Continuous bioaugmentation (low and high): BO (5 mg/kg) and inoculating strains [2×10^6 (low) or 2×10^8 (high) CFU/g soil] were added repeatedly every three days for 10 times. The data are presented as mean values ± SD (n = 3 biological independent replicates). Source data are provided as a Source Data file.

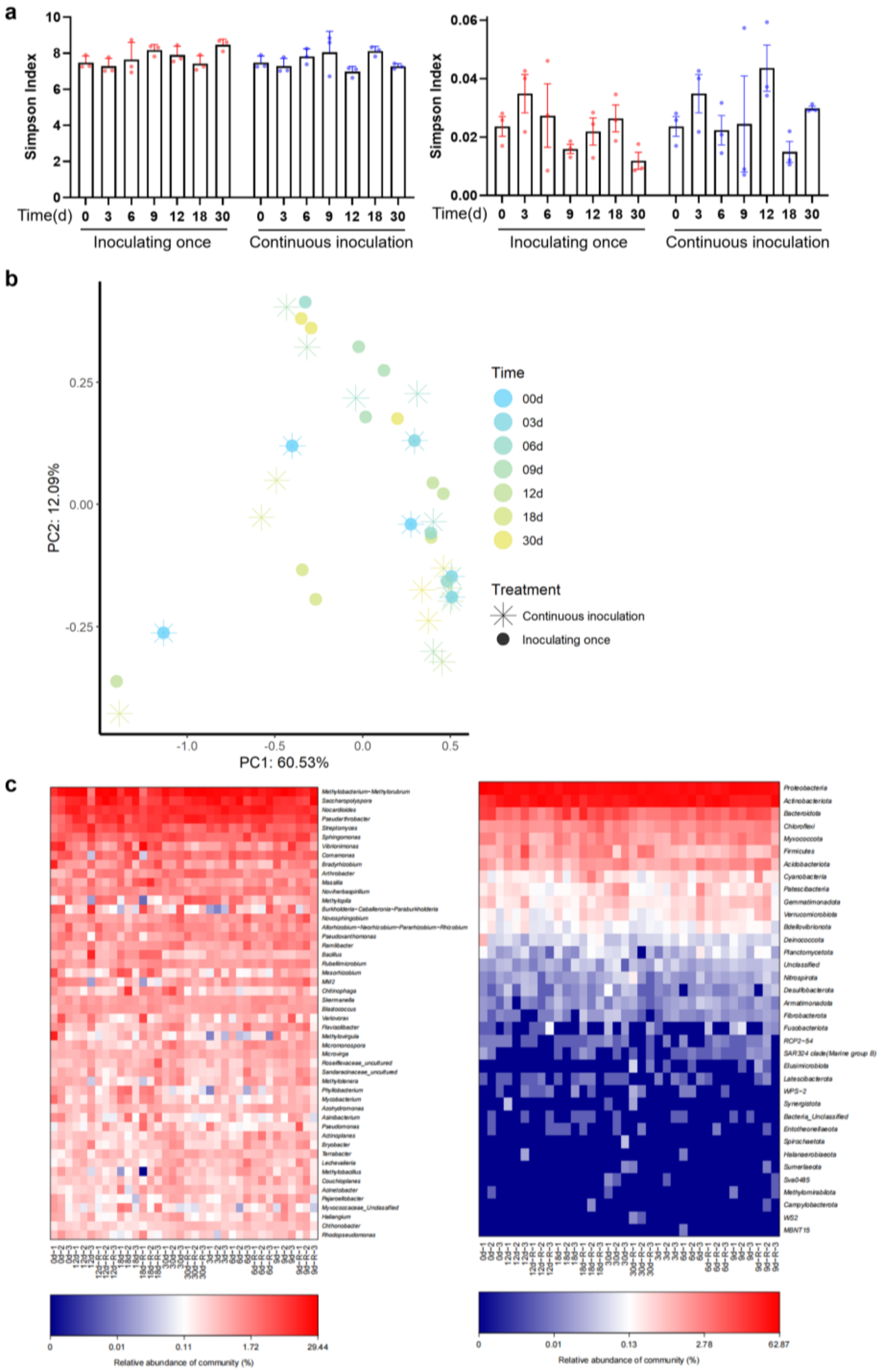

Supplementary Fig. 3. Microbial diversity and community composition during domestication with different treatments. **a** α-diversity of the soil microbiomes. The data are presented as mean values ± SD (n = 3 biological independent replicates). Inoculating once, inoculating two strains X-1 and 7D-2 at a ratio of 1:1, with a final concentration of approximately 2 × 10^6 CFU/g soil for each strain, and adding 5 mg/kg of bromoxynil octanoate (BO) only once; Continuous inoculation, BO (5 mg/kg) and inoculating strains (2 × 10^6 CFU/g soil) were added repeatedly every three days for 10 times. **b** Principal coordinates analysis (PCoA) with Bray–Curtis distances of bacterial communities. No significant clusters were detected for treatments and times. **c** Heatmaps showing relative abundances of genera (left) and phyla (right) identified in soils with different treatments. No significant difference was detected among treatments and times. Source data are provided as a Source Data file.

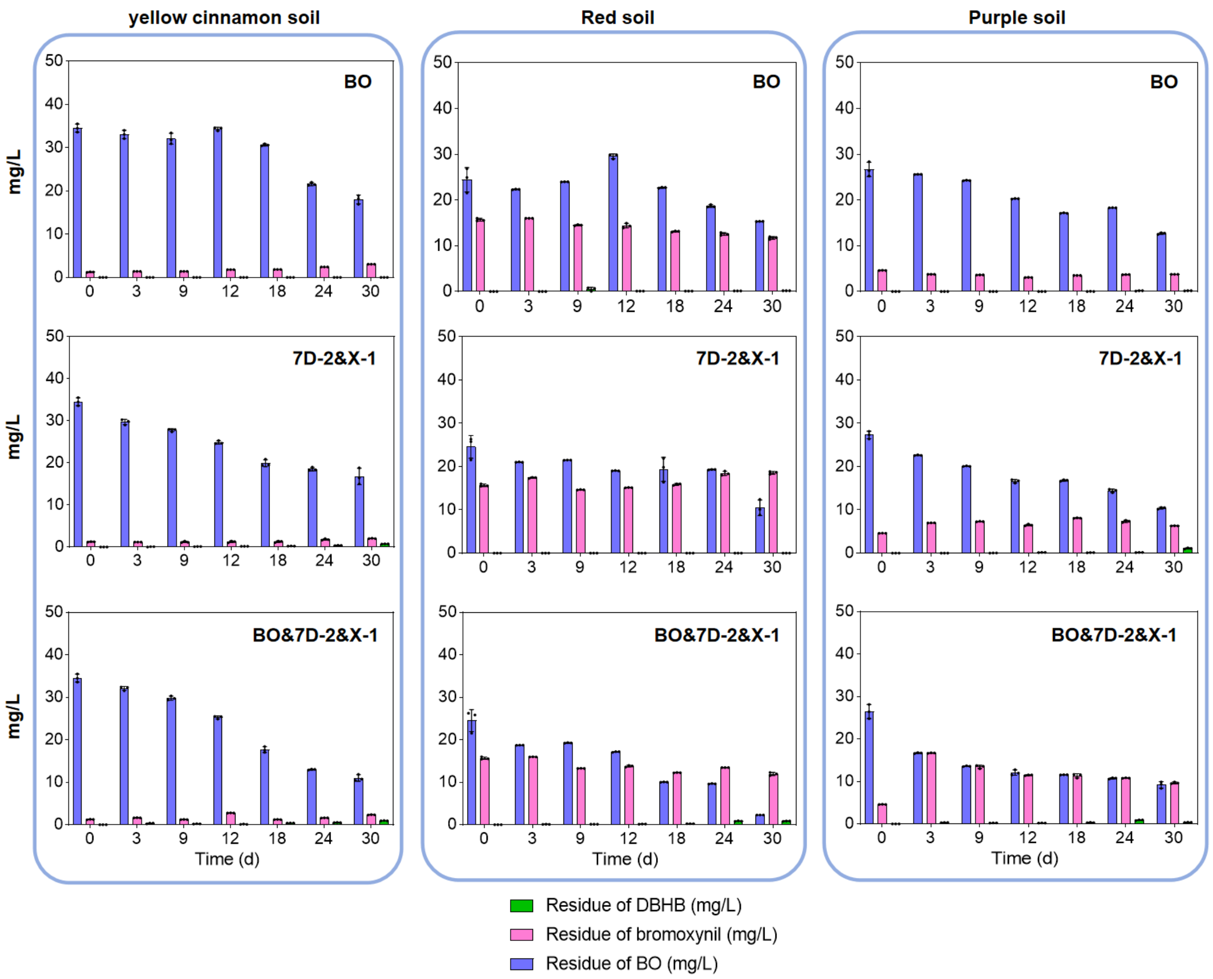

Supplementary Fig. 4. Herbicide residues in soil samples of BO groups. Blue bars represent the residue of BO. Pink bars represent the residue of bromoxynil. Green bars represent the residue of DBHB. P, purple soil; Y, yellow cinnamon soil; R, red soil. 1, 2, and 3 represent treatments with BO, syntrophic consortium (7D-2&X-1), and combination of BO and syntrophic consortium (BO&7D-2&X-1), respectively. BO, bromoxynil octanoate; DBHB, 3,5-dibromo-4-hydroxybenzoate. The data are presented as mean values ± SD (n = 3 biological independent replicates). Source data are provided as a Source Data file.

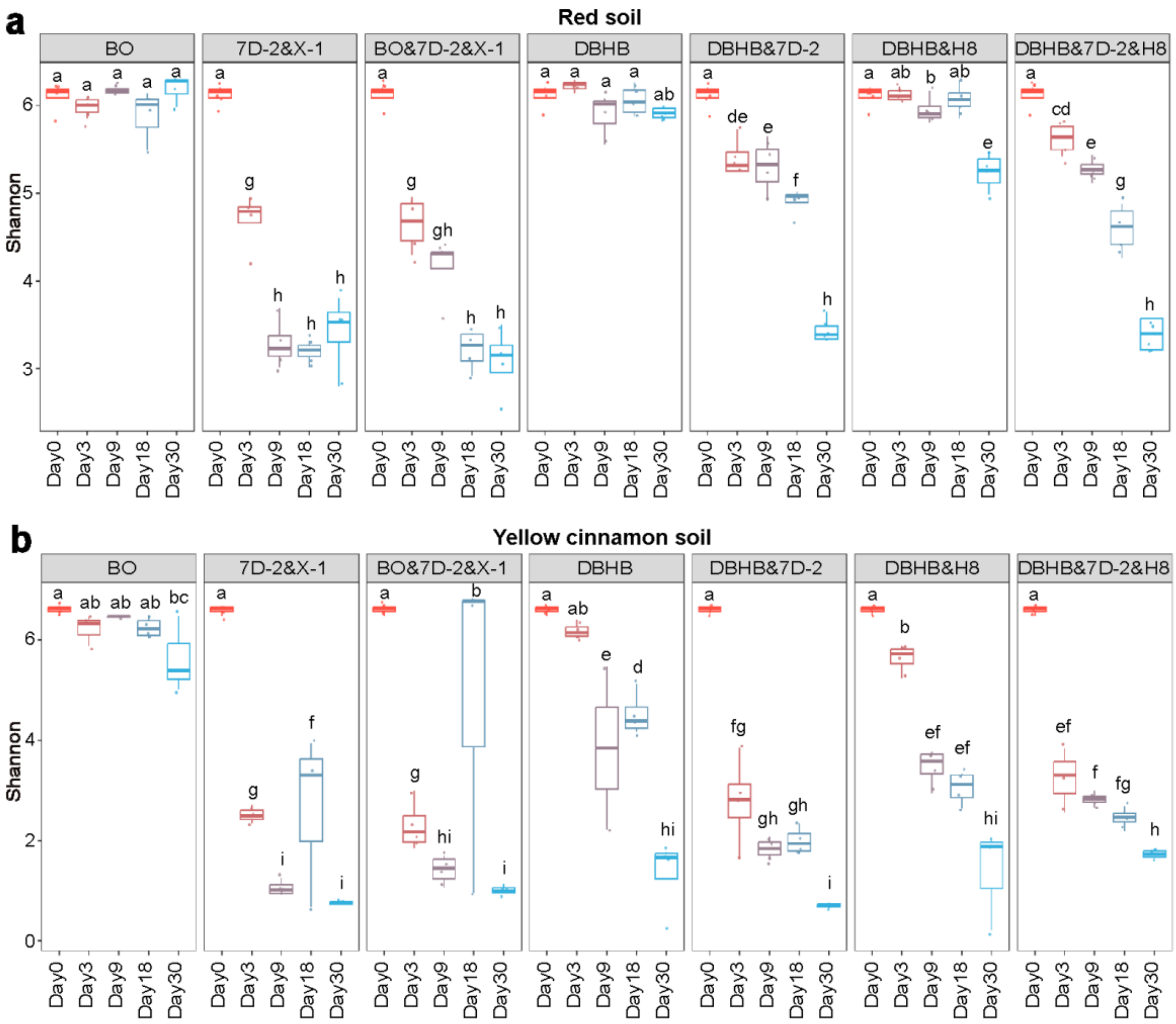

Supplementary Fig. 5. α-diversity levels of the soil microbiomes. **a** Microbiomes from red soils. **b** Microbiomes from yellow cinnamon soils. The boxplots show the Shannon indices of the three different soils with different treatments ($n = 4$ biological independent replicates). The letters above the boxplots show significant differences between samples at $P < 0.05$ (one-way ANOVA with correction by Tukey's HSD test). The horizontal bars within the boxes represent the medians. The tops and bottoms of the boxes represent the 75th and 25th quartiles, respectively. BO, bromoxynil octanoate; DBHB, 3,5-dibromo-4-hydroxybenzoate. Source data are provided as a Source Data file.

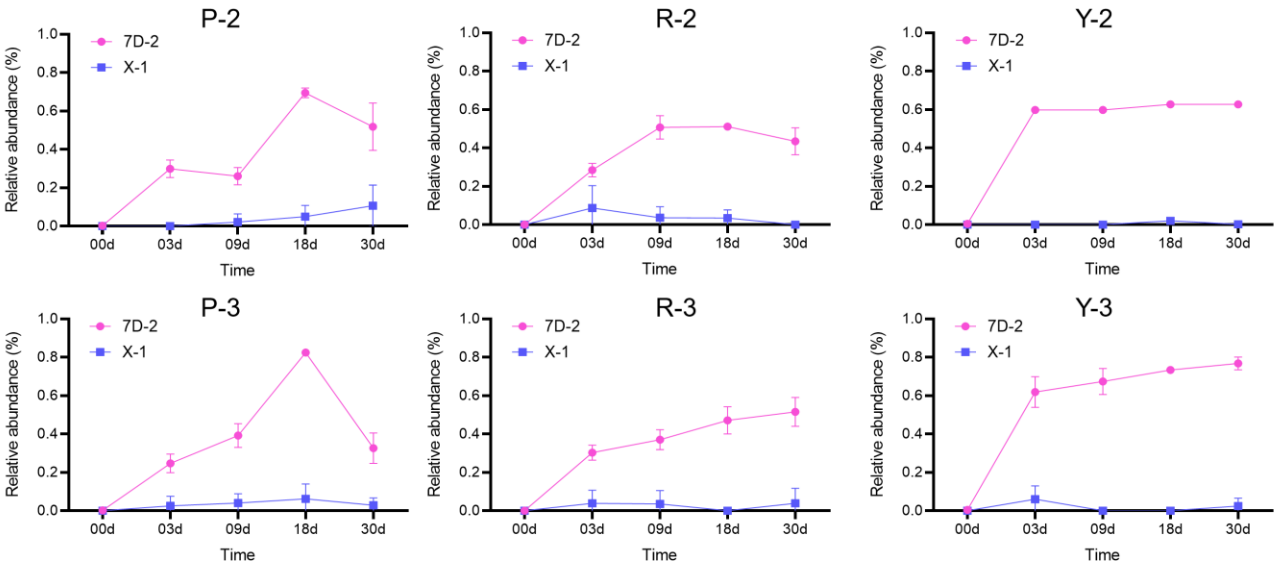

Supplementary Fig. 6. The relative abundances of strain 7D-2 and X-1 in treatments of inoculation. P, purple soil; Y, yellow cinnamon soil; R, red soil. -2 and -3 represent treatments with inoculation of the synergistic consortium (7D-2&X-1), and the combination of bromoxynil octanoate (BO) and inoculation of the synergistic consortium (BO&7D-2&X-1), respectively. The data are presented as mean values ± SD (n = 4 biological independent replicates). Source data are provided as a Source Data file.

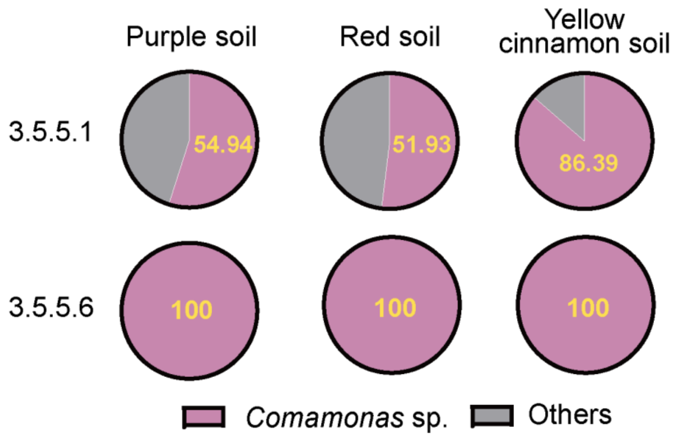

Supplementary Fig. 7. The taxonomic distribution of key enzymes involved in bromoxynil octanoate (BO) degradation in treated microbiome from Day 30 samples (n = 3 biological independent replicates). The nitrilase (EC 3.5.5.1 and EC 3.5.5.6) involves in the transformation from bromoxynil to 3,5-dibromo-4-hydroxybenzoate (DBHB), which is the key reaction for BO degradation. The result showed most of the bromoxynil degradation was driven by the inoculated bacteria (i.e. *Comamonas* sp. 7D-2), especially for the yellow cinnamon soil. Source data are provided as a Source Data file.

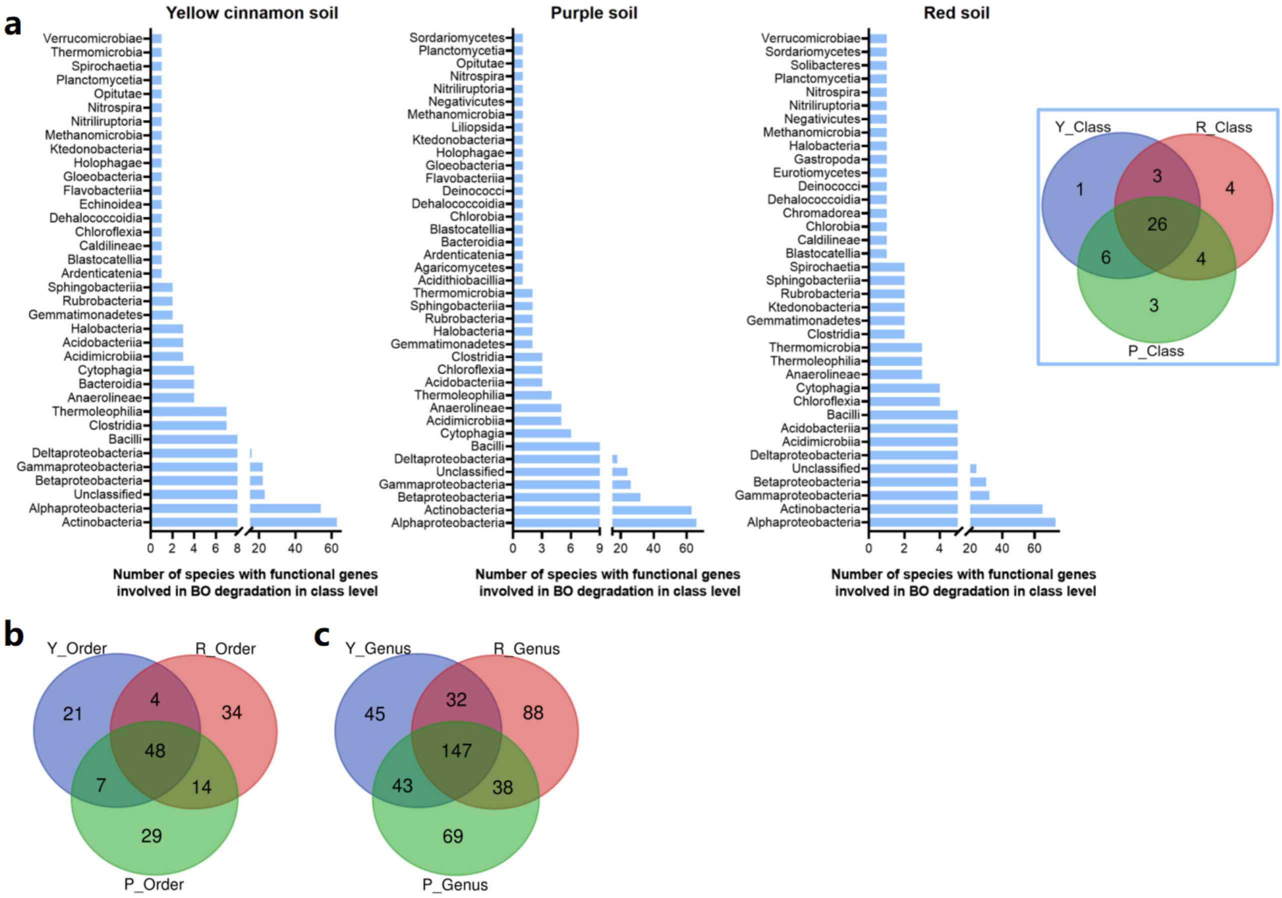

Supplementary Fig. 8. Taxonomic classification of the functional genes involved in bromoxynil octanoate (BO) degradation at the order (**a**), class (**b**), and genus (**c**) level. The bar and venn diagrams show taxonomic classification of the functional genes involved in BO degradation as shown in Fig.4b. The results indicate that bacteria with the functional genes involved in BO degradation were shared among different soils after treatments. Source data are provided as a Source Data file.

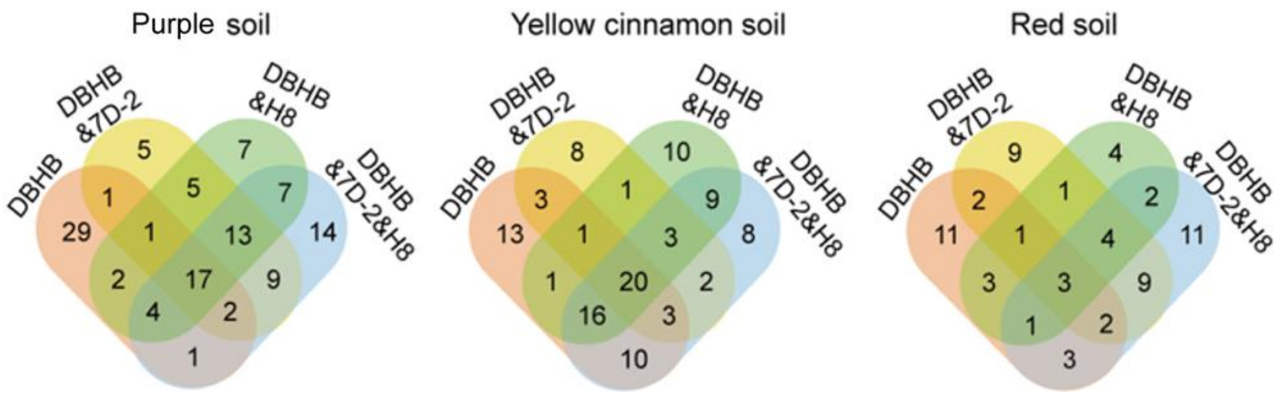

Supplementary Fig. 9. Venn diagram of differentially abundant genera identified by LEfSe analysis for 3,5-dibromo-4-hydroxybenzoate (DBHB) group (n = 12 biological independent replicates for early phase; n = 8 biological independent replicates for late phase). Source data are provided as a Source Data file.

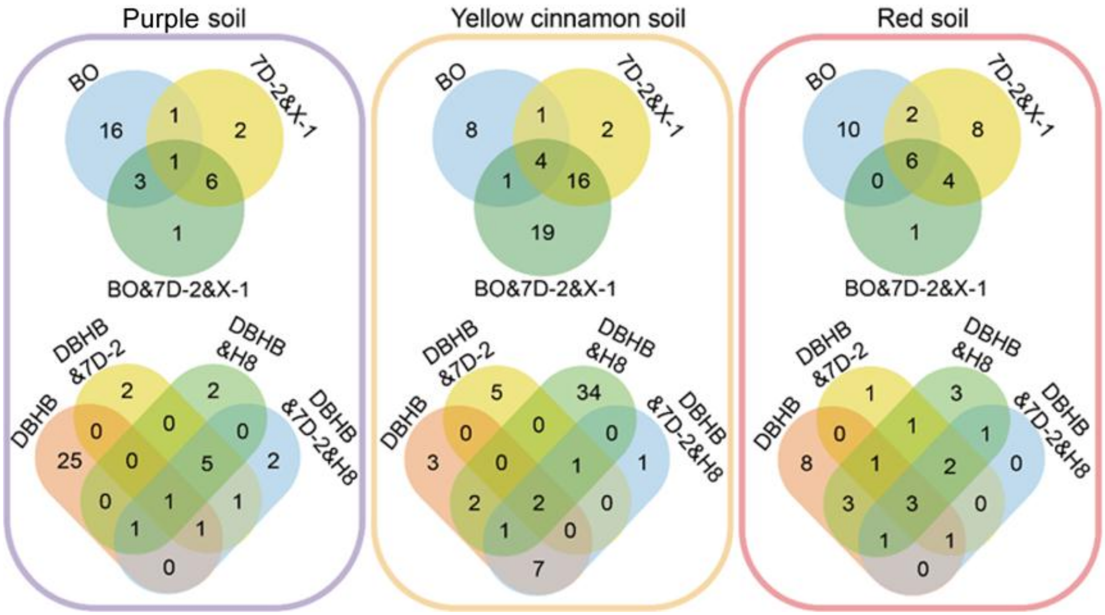

Supplementary Fig. 10. Venn diagram of genera with increased abundances in late phase compared to early phase identified by LEfSe analysis (n = 12 biological independent replicates for early phase; n = 8 biological independent replicates for late phase). Early phase, 0-9 days; Late phase, 18-30 days. BO, bromoxynil octanoate; DBHB, 3,5-dibromo-4-hydroxybenzoate. Source data are provided as a Source Data file.

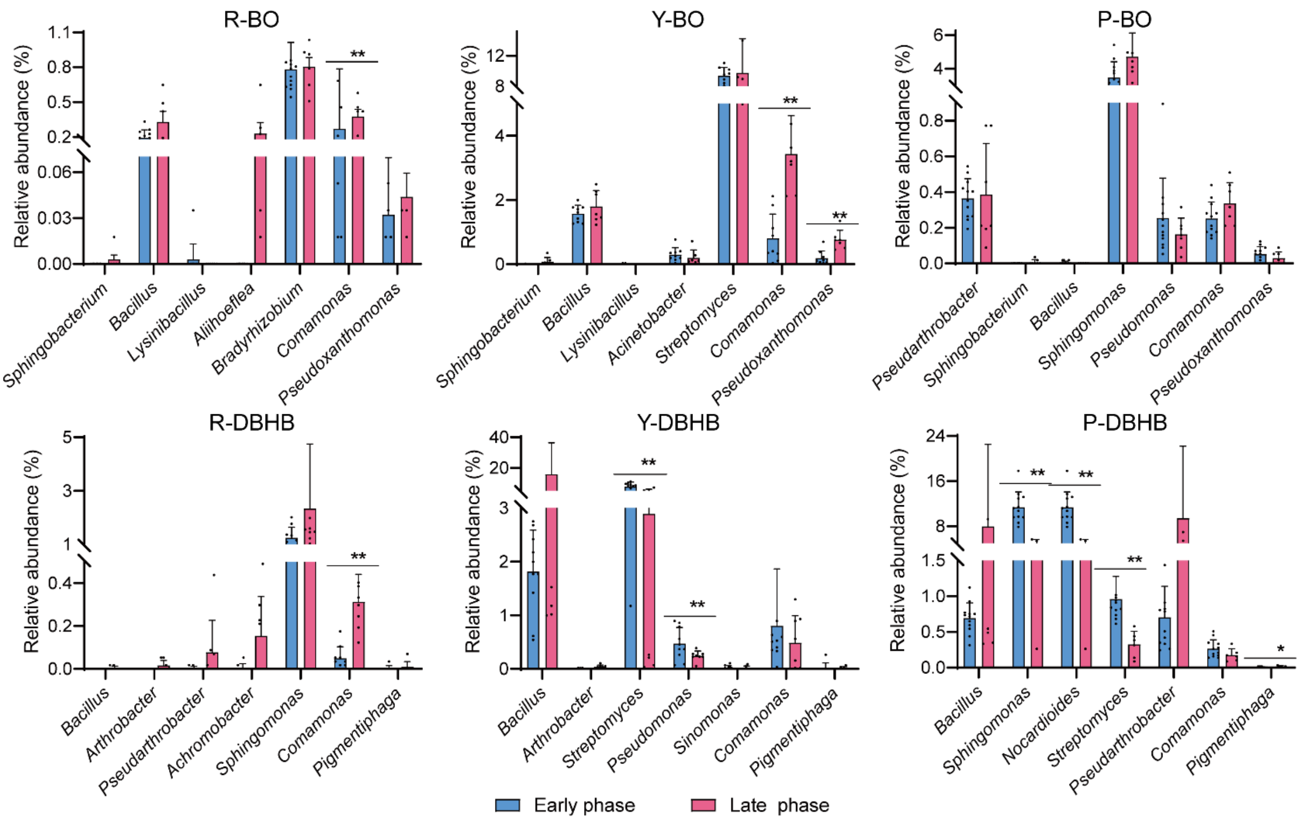

Supplementary Fig. 11. Relative abundances of 18 keystone genera in in soils only inoculated with herbicides. The average abundances of each keystone genus in samples at Days 0-9 (early phase) and Days 18-30 (late phase) are shown. The data are presented as mean values ± SD (n = 12 biological independent replicates for early phase; n = 8 biological independent replicates for late phase). The significance of differences was assessed using a two-sided Student's t-test (**P < 0.01; *P < 0.05). P, purple soil; Y, yellow cinnamon soil; R, red soil. BO, bromoxynil octanoate; DBHB, 3,5-dibromo-4-hydroxybenzoate. Source data are provided as a Source Data file.

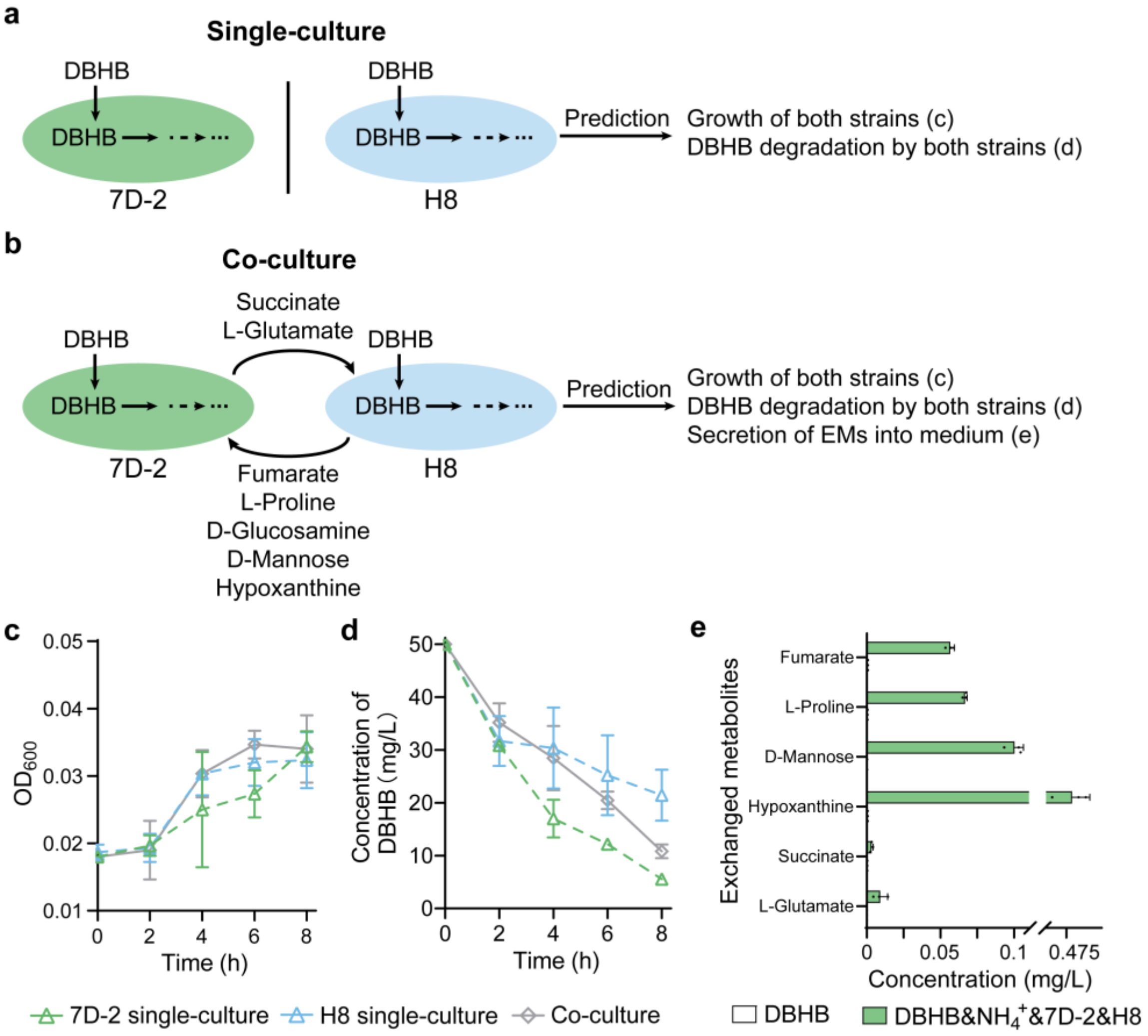

Supplementary Fig. 12. Simulations and experimental validations of metabolic interactions between strain 7D-2 and H8. **a, b** Predicted metabolic interactions between strains 7D-2 and H8 in single-culture (a) and co-culture (b) by community modeling. Five testable predictions were provided for experimental validation of the simulations. The letters in parentheses followed the predictions refers to the panels (c-e) supporting the corresponding predictions. **c** The cell growth of 7D-2 and H8 growing in single-culture versus co-culture. The log transformed colony-forming unit (CFU) of each strain cultured in medium with 3,5-dibromo-4-hydroxybenzoate (DBHB) as the sole carbon source is showed. **d** The residues of DBHB during DBHB degradation by individual strains of 7D-2 and H8 or co-cultures. **e** Identification of the predicted exchanged metabolites via LC–MS in co-culture of strains 7D-2 and H8. Medium with DBHB and NH_4^+ as the carbon and nitrogen sources was used for both simulation and experimental validation. For c-e, the data are presented as mean values ± SD (n = 3 biological independent replicates). Source data are provided as a Source Data file.

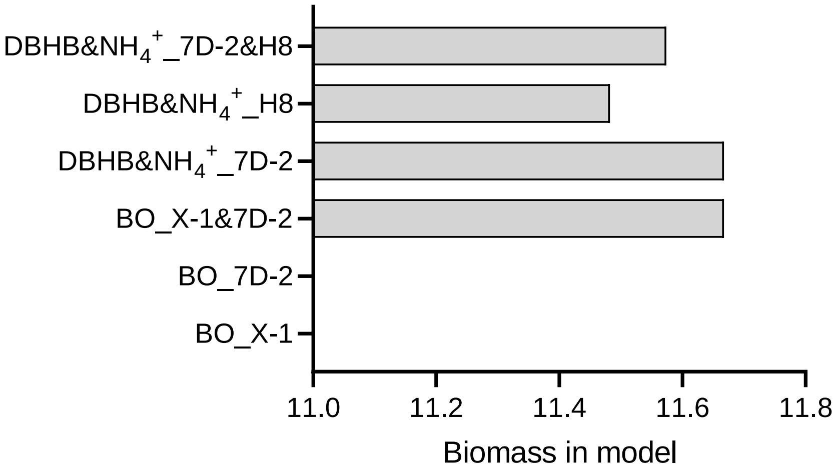

Supplementary Fig. 13. Simulations of the biomass of strain X-1, 7D-2 or H8 growing separately versus in co-cultures (X-1&7D-2 or 7D-2&H8). The simulations were performed in the BO or DBHB&NH_4^+ medium. BO, bromoxynil octanoate; DBHB, 3,5-dibromo-4-hydroxybenzoate. Source data are provided as a Source Data file.

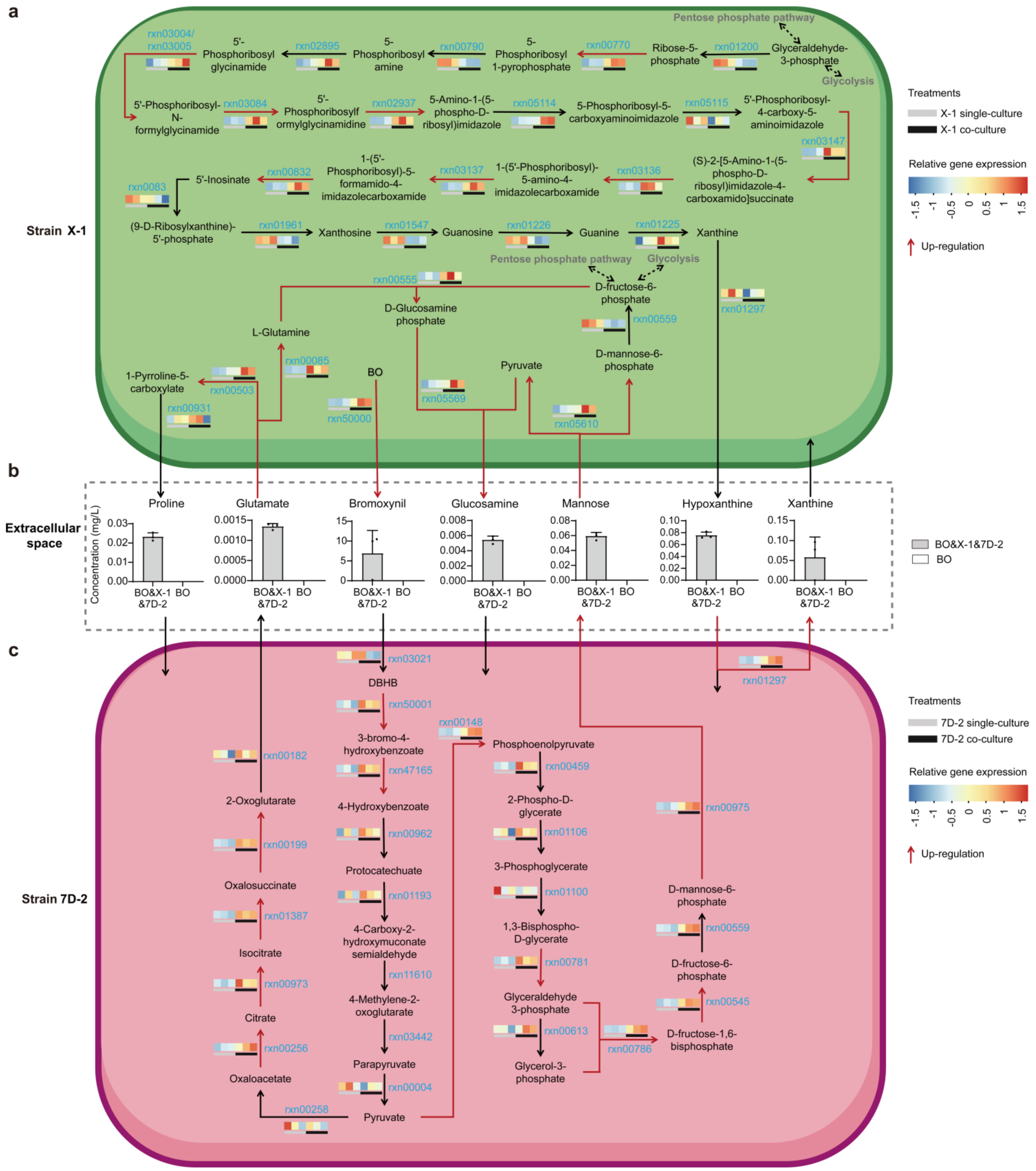

Supplementary Fig. 14. Simplified metabolic network depicting metabolic interactions between strains X-1 and 7D-2. **a, c** The predicted reactions active in the strains X-1 (a) and 7D-2 (c). The codes starting with "rxn" represent metabolic reactions. Supplementary Table 7 provides detailed information on these reactions, including reactants, products, stoichiometry, direction, ΔG, and genes coding the enzymes required for the metabolic interactions. The heatmap shows gene expression levels of strains X-1 and 7D-2 in single-culture and co-culture, and each treatment had three replicates ($n = 3$ biological independent replicates). Red indicates increased transcript abundance, while blue indicates decreased transcript abundance. The red arrows indicate up-regulated genes in

co-cultures compared to single-cultures. **b** Prediction and experimental validation of exchanged metabolites between strains 7D-2 and X-1. The data are presented as mean values ± SD (n = 3 biological independent replicates). The bar graph displays the concentration of each exchanged metabolite detected by LC–MS in the medium of co-cultures (BO&X-1&7D-2). Medium with bromoxynil octanoate (BO) as the only carbon and nitrogen source was used for both simulation and experimental validation. The medium without inoculation of any strains was used as control. Notably, these exchanged metabolites were not initially present in the medium, and their detection validates the secretion of these metabolites by the strains. Source data are provided as a Source Data file.

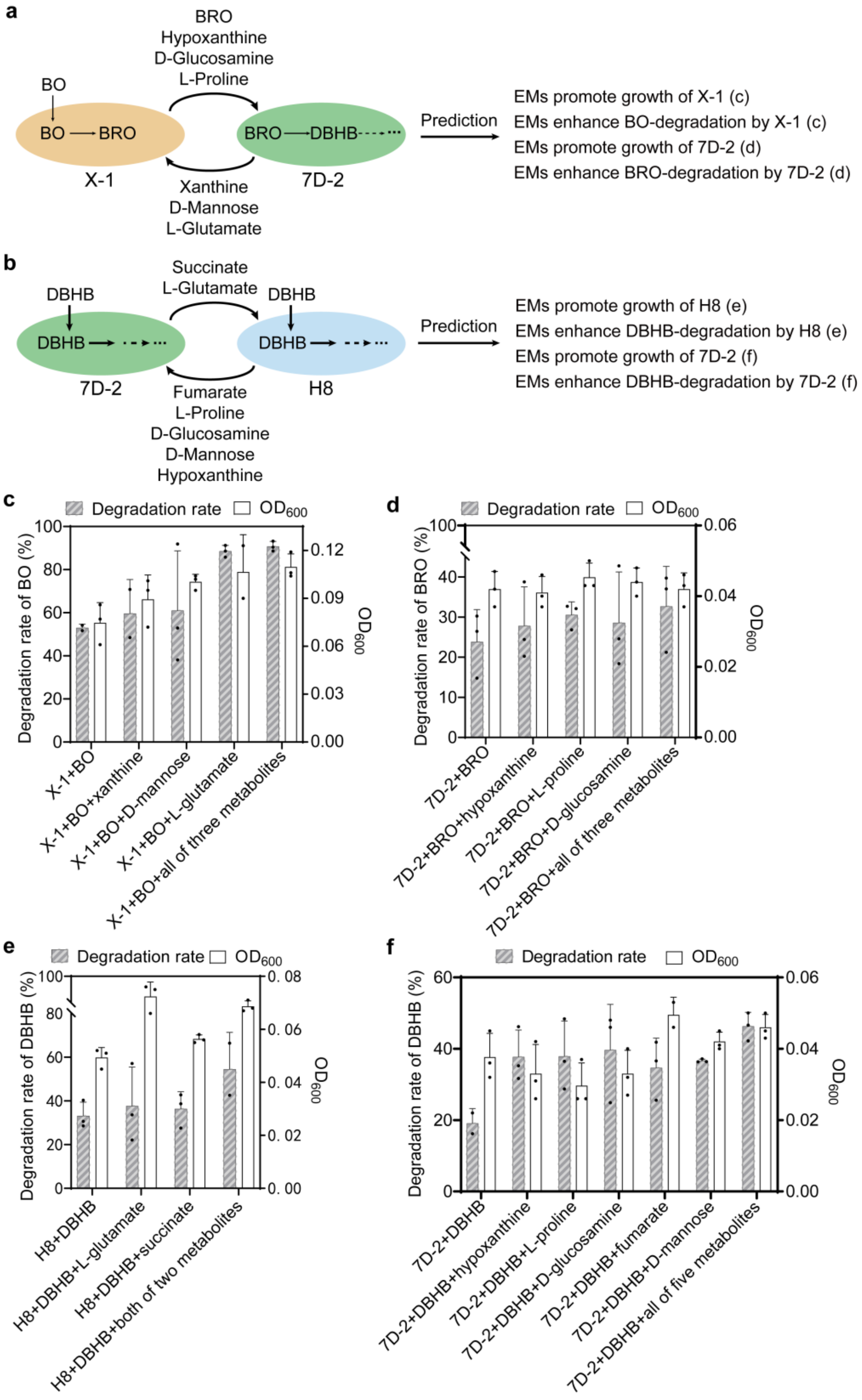

Supplementary Fig. 15. Experimental validations of the function of exchanged metabolites (EMs) in two-member consortia. **a, b** Predicted metabolic interactions in 7D-2&X-1 (a) and 7D-2&H8 (b) by modeling. Eight testable predictions about the function of exchanged metabolites were provided for experimental validation (**c-f**). The bar plots show the growth and fraction of BO/DBHB left in medium containing BO/DBHB as a sole nitrogen and carbon source versus the same medium supplemented by exchange metabolites. The growth and degradation were enhanced in the supplemented medium for strains X-1, 7D-2, and H8, indicating the exchanges could be additional

carbon and/or nitrogen source for degraders which increased the biomass of degrader and finally improve the degradation. BO, bromoxynil octanoate; DBHB, 3,5-dibromo-4-hydroxybenzoate; BRO, bromoxynil. The data are presented as mean values ± SD ($n = 3$ biological independent replicates). Source data are provided as a Source Data file.

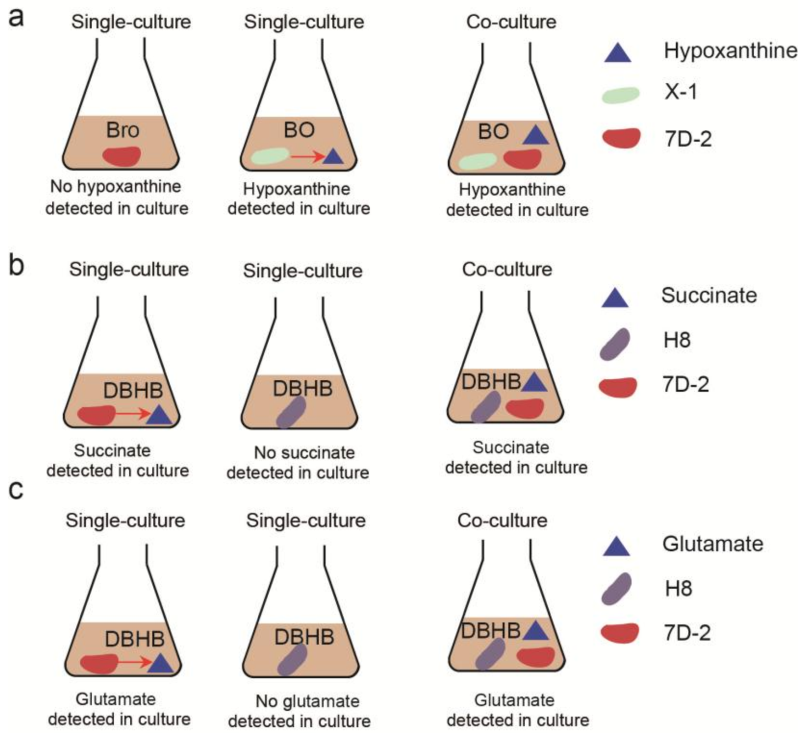

Supplementary Fig. 16. LC-MS analysis of the exchanged metabolites in single-cultures and co-cultures. **a** Secretion of hypoxanthine by X-1. **b** Secretion of succinate by 7D-2. **c** Secretion of glutamate by 7D-2. For single-cultures, the strains X-1 and H8 were cultured in the MM medium containing BO and DBHB respectively, while the strain 7D-2 was cultured in the MM medium containing Bro or DBHB. The co-cultures of X-1&7D-2 and 7D-2&H8 were cultured in the MM medium containing BO and DBHB respectively. All final cultures were tested after 4 hours of culture. BO, bromoxynil octanoate; Bro, bromoxynil; DBHB, 3,5-dibromo-4-hydroxybenzoate.

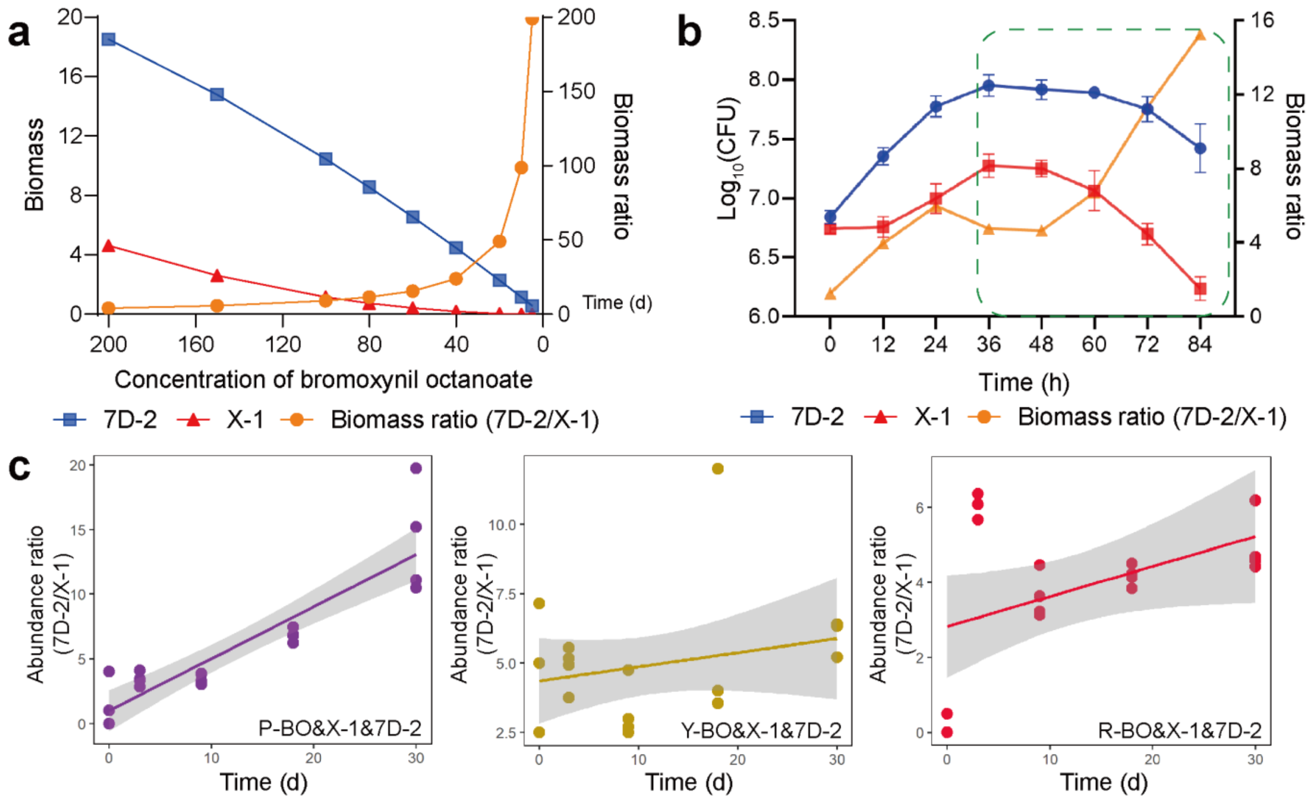

Supplementary Fig. 17. Simulations and experimental validations of metabolic interactions in two-member consortia. **a, b** Predicted changes in the biomass ratio of strains 7D-2 and X-1 according to bromoxynil octanoate (BO) content in the medium (**a**) and experimental validation (**b**), showing that the biomass ratio of the two strains increased with decreasing BO content. For b, the data are presented as mean values ± SD (n = 3 biological independent replicates). **c** The biomass ratios of 7D-2 and X-1 in soils treated with BO-7D-2&X-1 increased over time, consistent with the prediction (n = 4 biological independent replicates). P, purple soil; Y, yellow cinnamon soil; R, red soil. Linear regression line is indicated by the colored line. The 95% confidence interval of the linear regression line is indicated by gray bands. P values are two-sided. Source data are provided as a Source Data file.

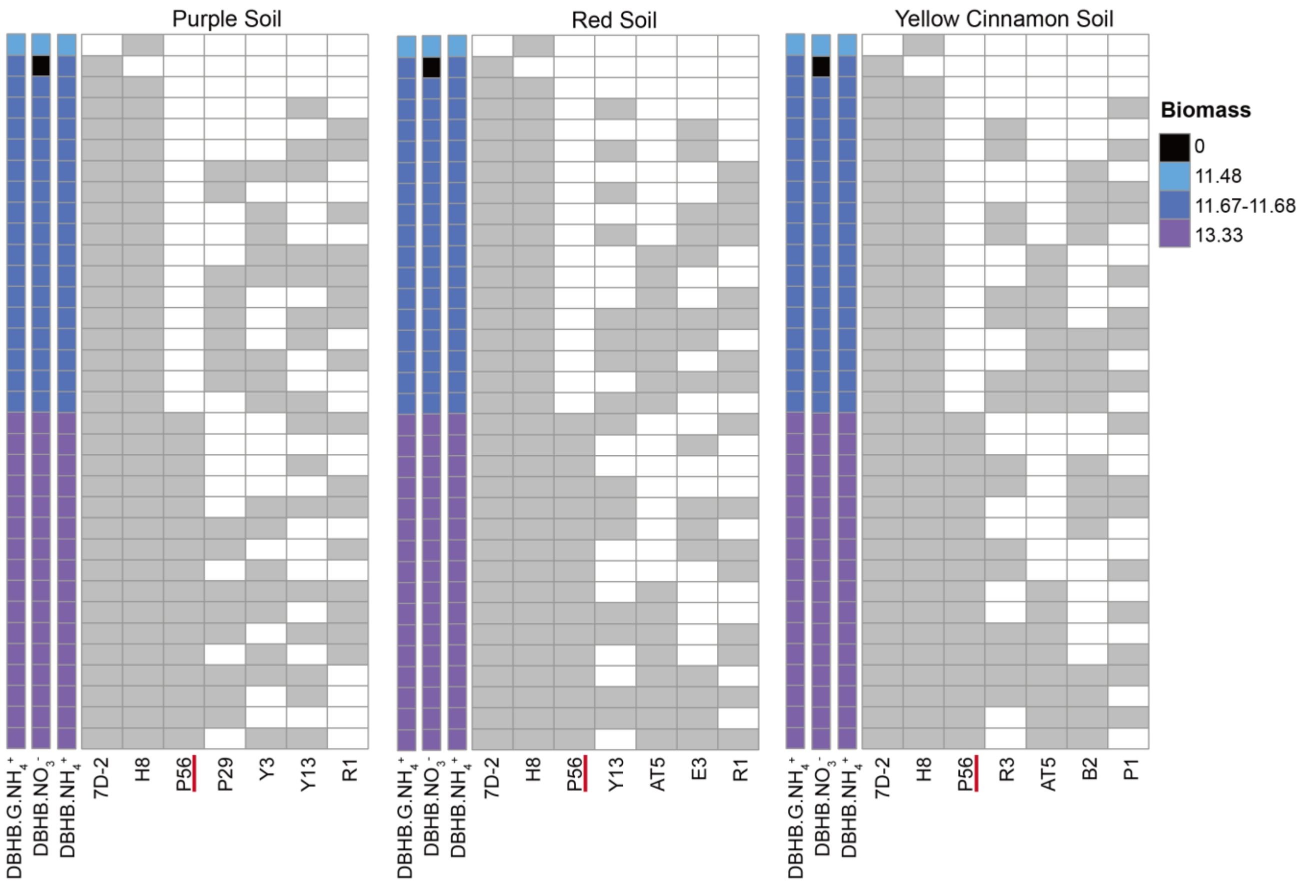

Supplementary Fig. 18. Predicted biomass of bacterial combinations by SuperCC. The grey/white cells in grids indicate species included/not included in the bacterial combination, respectively. The bars on the left of the grids are indicative of biomass predicted in three media: DBHB medium supplemented with NH_4^+ (DBHB.NH_4^+) as the sole carbon and nitrogen source, DBHB medium supplemented with NO_3^- (DBHB.NO_3^-), and DBHB medium supplemented with glucose and NH_4^+ (DBHB.G.NH_4^+). DBHB, 3,5-dibromo-4-hydroxybenzoate. Source data are provided as a Source Data file.

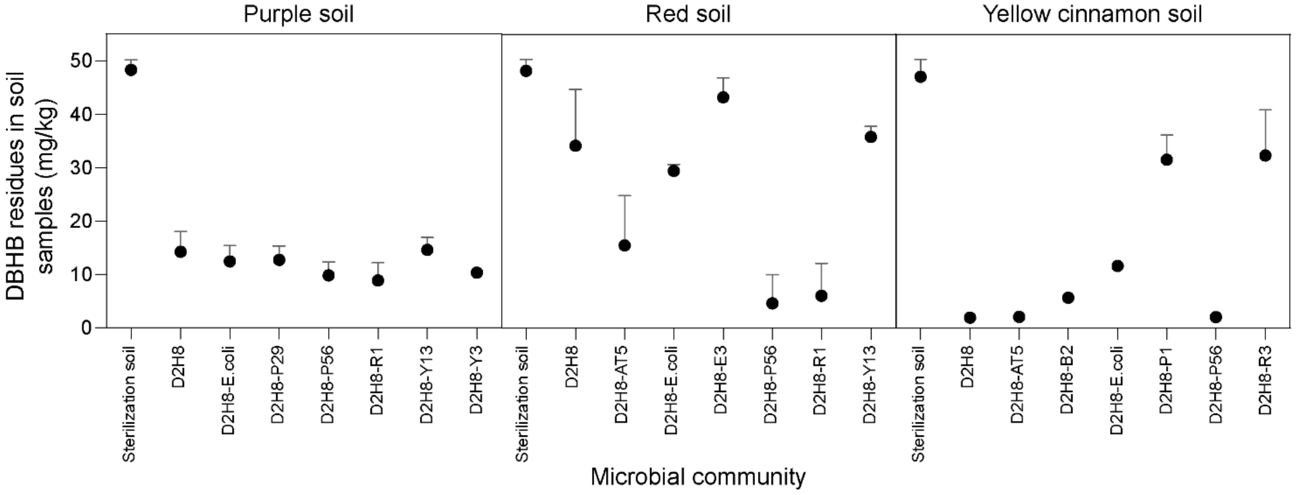

Supplementary Fig. 19. Experimental validation of simulations of DBHB treatments by pot experiments with three-member consortia in three soils separately. All three-member consortia include strains 7D-2 and H8 together with one selected keystone. The consortia including strains 7D-2, H8 and an exogenous strain (*Escherichia coli*) were set as negative control. DBHB, 3,5-dibromo-4-hydroxybenzoate. The data are presented as mean values ± SD (n = 3 biological independent replicates). Source data are provided as a Source Data file.

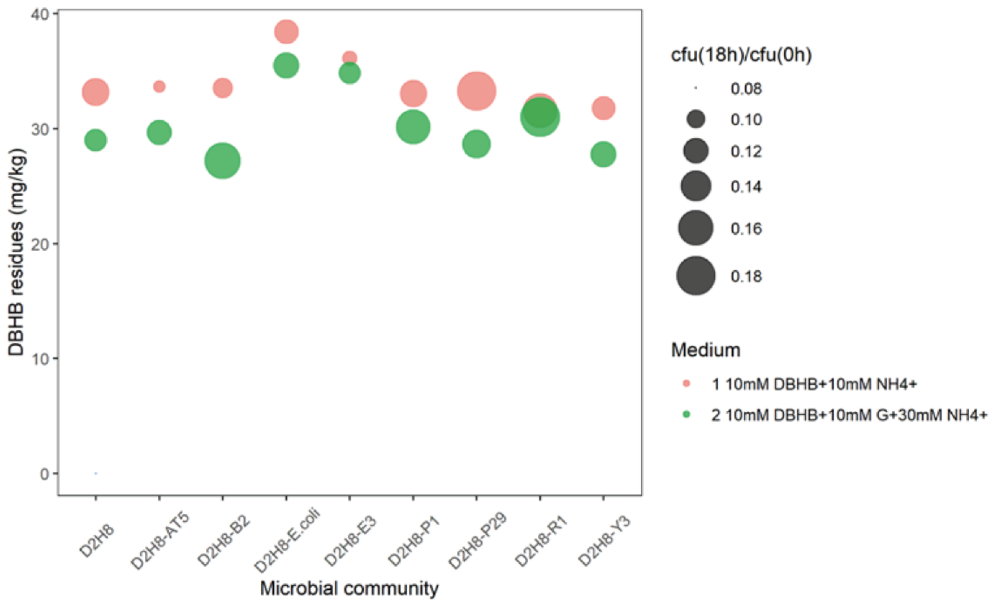

Supplementary Fig. 20. Experimental validations of DBHB degradation and bacterial growth performances in two media. DBHB, 3,5-dibromo-4-hydroxybenzoate. The color of dots represents the medium and the size represents the growth of the synthetic consortia. Source data are provided as a Source Data file.

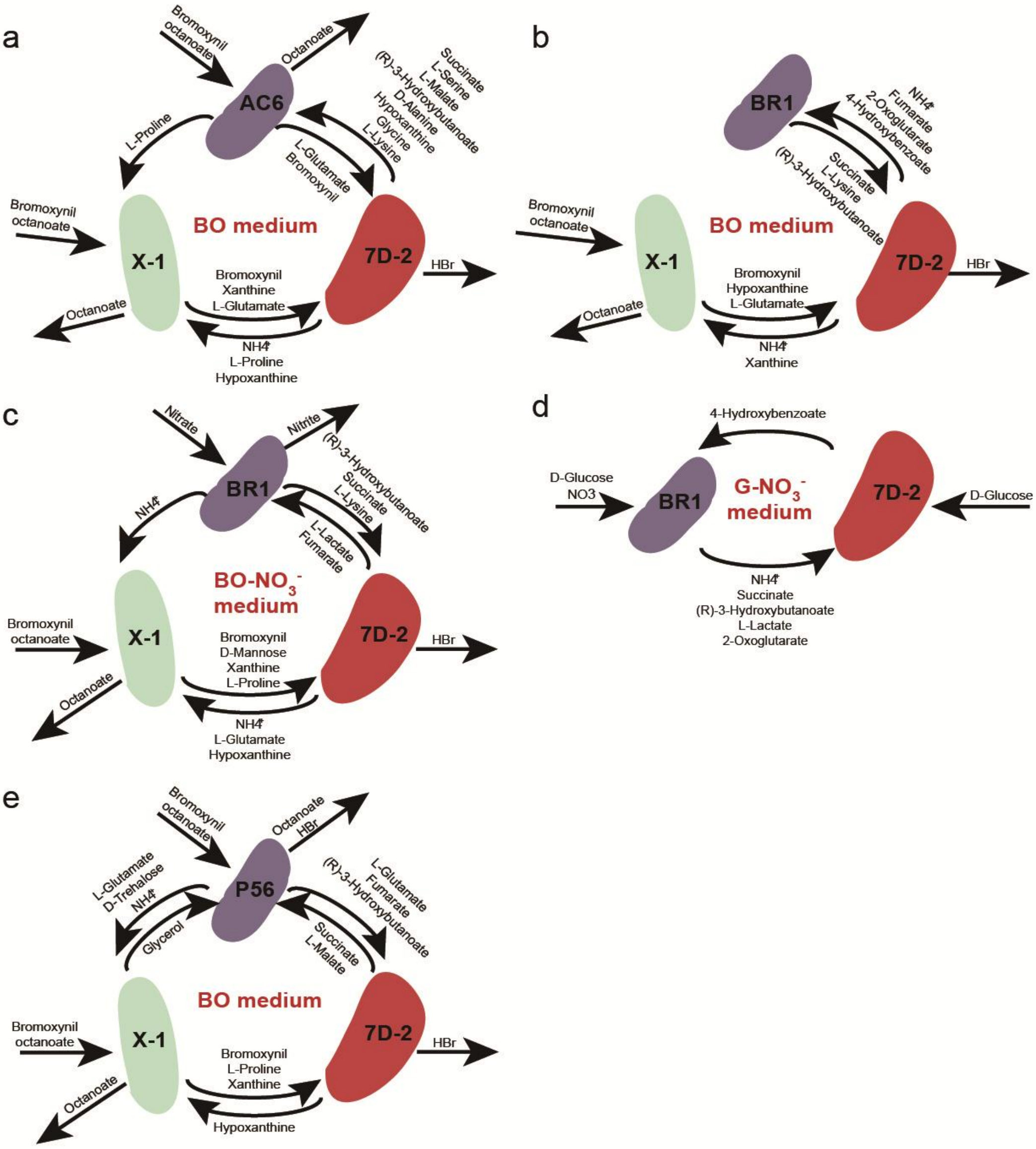

Supplementary Fig. 21. Simulations of bacterial interactions among synthetic consortia by SuperCC. **a** Strains X-1, 7D-2, and AC6 simulated in BO medium. BO, bromoxynil octanoate. **b** Strains X-1, 7D-2, and BR1 simulated in BO medium. **c** Strains X-1, 7D-2, and BR1 simulated in BO-NO_3^- medium. **d** Strains 7D-2 and BR1 simulated in G-NO_3^- medium. **e** Strains X-1, 7D-2, and P56 simulated in BO medium.

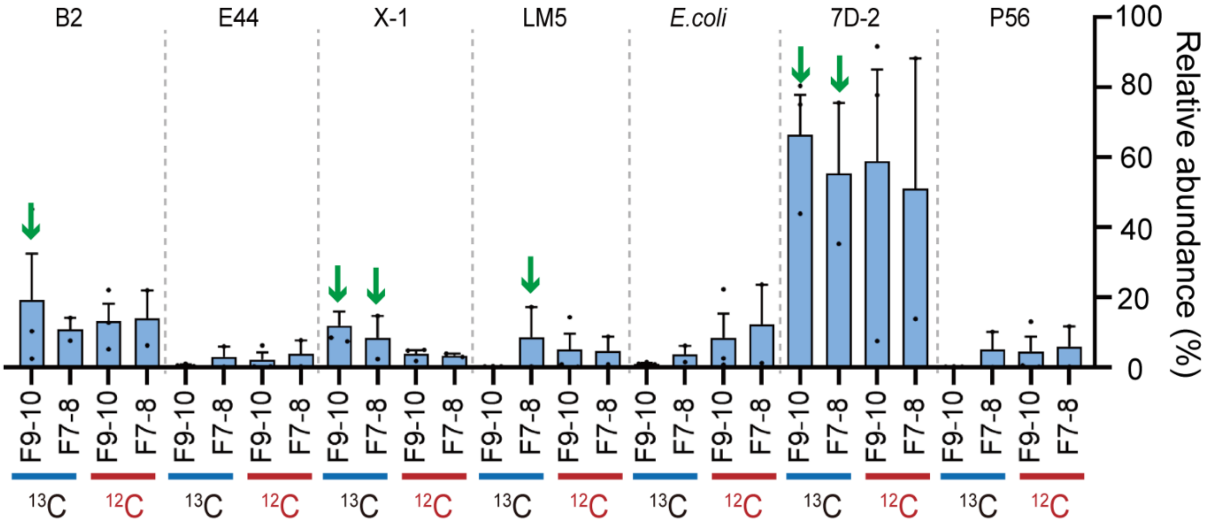

Supplementary Fig. 22. Relative abundances of ASVs in fraction 7 to 10 of normal 4-hydroxybenzoic acid and ^{13}C-labled 4-hydroxybenzoic acid samples after fractionation. The data are presented as mean values ± SD (n = 3 biological independent replicates). Source data are provided as a Source Data file.

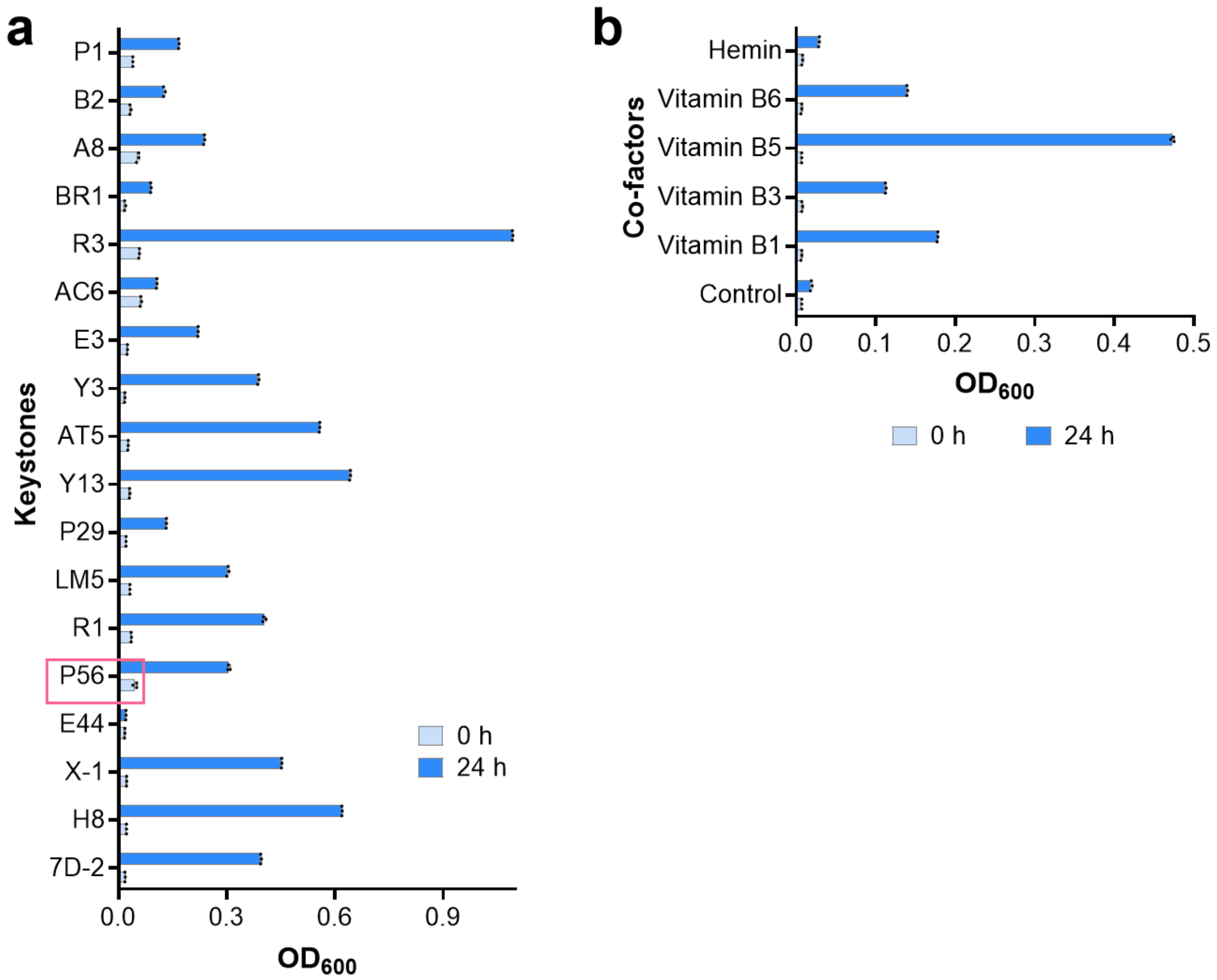

Supplementary Fig. 23. The cell growth of keystones in minimal mineral medium (MM medium) with glucose and NH_4Cl sources and co-factors separately. **a** The cell growth of keystones in glucose-NH_4Cl-MM medium. **b** The cell growth of strain E44 in glucose-NH_4Cl-MM medium with different co-factors. Control, no additional co-factors. The data are presented as mean values ± SD (n = 3 biological independent replicates). Source data are provided as a Source Data file.

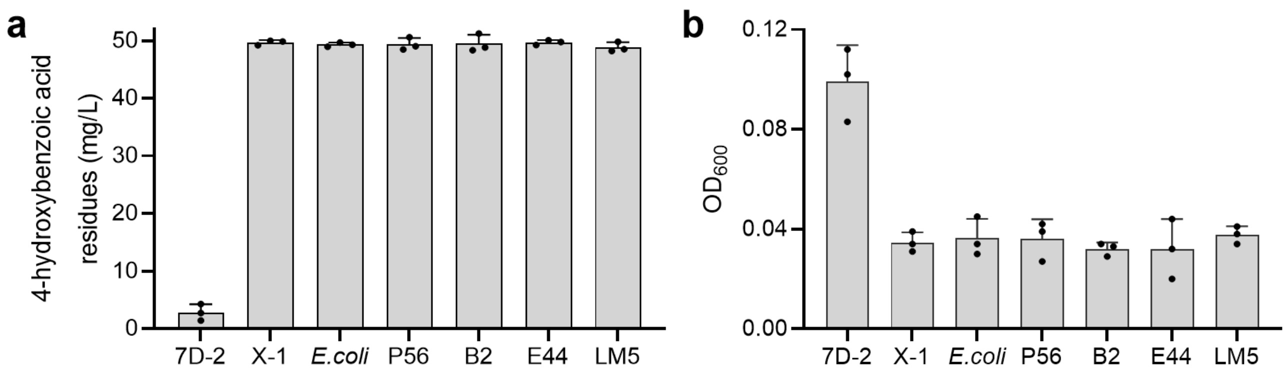

Supplementary Fig. 24. The degradation ability (**a**) and growth (**b**) of 7 stains in the MM medium supplemented with 4-hydroxybenzoic acid as carbon source. The data are presented as mean values ± SD (n = 3 biological independent replicates). Source data are provided as a Source Data file.

Supplementary Tables

Supplementary Table 1. Information of 290 strains isolated from the in-situ soil.

**Isolates from red soil**	**Isolates from yellow cinnamon soil**	**Isolates from purple soil**
Achromobacter marplatensis E31	*Achromobacter marplatensis* NewY27	*Achromobacter insuavis* P72
Achromobacter marplatensis E43	*Achromobacter marplatensis* NewY36	*Aromatoleum diolicum* P13
Achromobacter marplatensis NewR28	*Achromobacter marplatensis* NewY44	*Arthrobacter ginsengisoli* P-2-33
Achromobacter pulmonis E38	*Achromobacter marplatensis* NewY58	*Arthrobacter ginsengisoli* P-2-34
Achromobacter pulmonis E39	*Achromobacter pulmonis* NewY1	*Azospirillum brasilense* P-2-32
Achromobacter pulmonis E46	*Achromobacter pulmonis* NewY10	*Bacillus albus* P-3-8
Achromobacter pulmonis NewR10	*Achromobacter pulmonis* NewY2	*Bacillus altitudinis* P15
Achromobacter pulmonis NewR22	*Achromobacter pulmonis* NewY31	*Bacillus aryabhattai* P82
Achromobacter pulmonis NewR31	*Achromobacter pulmonis* NewY4	*Bacillus aryabhattai* P-1-46
Achromobacter pulmonis NewR8	*Achromobacter pulmonis* NewY49	*Bacillus aryabhattai* P-2-20
Achromobacter pulmonis OldE2	*Achromobacter pulmonis* NewY57	*Bacillus aryabhattai* P56
Achromobacter pulmonis OldE3	*Achromobacter ruhlandii* NewY32	*Bacillus cereus* P-1-8
Acinetobacter guillouiae AC6	*Achromobacter ruhlandii* NewY43	*Bacillus cereus* P-2-31
Acinetobacter guillouiae R71	*Achromobacter ruhlandii* NewY45	*Bacillus megaterium* P-1-31
Acinetobacter radioresistens R-3-31	*Achromobacter ruhlandii* NewY46	*Bacillus onubensis* P-1-41
Acinetobacter radioresistens R-3-33	*Achromobacter ruhlandii* NewY59	*Bacillus oryzisoli* P-1-50
Acinetobacter radioresistens R-3-34	*Arthrobacter nitrophenolicus* Y-1-32	*Bacillus pacificus* P-3-10
Acinetobacter radioresistens R-3-38	*Arthrobacter cupressi* Y53	*Bacillus safensis subsp.safensis* P5
Acinetobacter seifertii NewR18	*Arthrobacter cupressi* Y38	*Bacillus subterraneus* P79
Acinetobacter seifertii NewR21	*Bacillus acidiceler* NewY63	*Bacillus tequilensis* P16
Acinetobacter seifertii NewR4	*Bacillus altitudinis* NewY33	*Bacillus zanthoxyli* P-1-45
Arthrobacter ginsengisoli E18	*Bacillus altitudinis* NewY62	*Bacillus zanthoxyli* P-2-9-2
Arthrobacter oryzae E29	*Bacillus aryabhattai* Y21	*Brevibacillus parabrevis* P-3-33
Arthrobacter oryzae E5	*Bacillus aryabhattai* NewY7	*Brevibacterium epidermidis* P-2-1
Arthrobacter pokkalii NewR31	*Bacillus aryabhattai* NewY8	*Brevibacterium epidermidis* P-2-12
Arthrobacter ulcerisalmonis E13	*Bacillus cereus* NewY14	*Brevibacterium epidermidis* P-3-2
Arthrobacter ulcerisalmonis E40	*Bacillus cereus* NewY9	*Brevibacterium frigoritolerans* P-2-8
Bacillus altitudinis R2	*Bacillus drentensis* Y103	*Brevundimonas olei* P-3-11
Bacillus aryabhattai E16	*Bacillus megaterium* Y112	*Brevundimonas olei* P-3-9
Bacillus aryabhattai NewR11	*Bacillus megaterium* NewY12	*Burkholderia ubonensis* P55
Bacillus aryabhattai R-2-4	*Bacillus tequilensis* NewY101	*Cellulosimicrobium funkei* P-2-10
Bacillus aryabhattai R86	*Bacillus velezensis* NewY38	*Cellulosimicrobium funkei* P-2-2
Bacillus cereus R55	*Bacillus velezensis* NewY64	*Comamonas testosteroni* P-1-38

Bacillus cucumis NewR14	*Bacillus zanthoxyli* NewY39	*Comamonas testosteroni* P-3-35
Bacillus cucumis R42	*Bacillus zanthoxyli* NewY47	*Comamonas testosteroni* P61
Bacillus depressus R-3-5	*Bacillus altitudinis* Y111	*Cupriavidus necator* P58
Bacillus drentensis R107	*Bacillus aryabhattai* Y-20-1-1	*Dyella koreensis* P103
Bacillus drentensis R89	*Bacillus drentensis* Y79	*Ensifer mexicanus* P8
Bacillus megaterium R-1-12	*Bacillus megaterium* Y80	*Ensifer mexicanus* R7
Bacillus paramycoides E30	*Bacillus pseudomycoides* Y-2-44	*Fictibacillus arsenicus* P-3-31
Bacillus paramycoides E32	*Bacillus tequilensis* Y31	*Fictibacillus phosphorivorans* P7
Bacillus paramycoides E37	*Bacillus zanthoxyli* NewY29	*Hydrogenophaga intermedia* P11
Bacillus paramycoides E45	*Brevibacterium frigoritolerans* NewY6	*Lysobacter panacisoli* P-1-47
Bacillus paramycoides R3	*Brevibacterium epidermidis* Y-3-2	*Lysobacter panacisoli* P-1-49
Bacillus safensis subsp. safensis R103-2	*Brevibacterium sediminis* Y-3-18	*Mesobacillus subterraneus* P-2-36
Bacillus siamensis NewR-1-2	*Chryseobacterium endophyticum* NewY27	*Methylorubrum extorquens* P-2-5
Bacillus siamensis R22	*Comamonas testosteroni* Y-2-37	*Micrococcus aloeverae* P-1-42
Bacillus velezensis E17	*Comamonas testosteroni* Y-2-38	*Nocardioides aromaticivorans* P-1-5
Bacillus wiedmannii R110	*Comamonas testosteroni* Y-3-32	*Ochrobactrum anthropi* P-2-39
Bacillus wiedmannii R46	*Curtobacterium albidum* Y51	*Ochrobactrum intermedium* P-2-3
Bacillus wudalianchiensis R-3-3	*Ensifer meliloti* Y-1-35	*Ochrobactrum pseudintermedium* P-1-9
Bosea vestrisii R103	*Ensifer meliloti* Y-2-22	*Olivibacter soli* P-1-35
Brevibacillus brevis R-3-11	*Ensifer psoraleae* Y-2-25	*Olivibacter soli* P-1-37
Brevibacillus brevis R45	*Lysinibacillus xylanilyticus* NewY22	*Paenarthrobacter aurescens* P-3-36
Brevibacillus parabrevis R-3-32	*Massilia chloroacetimidivorans* Y3	*Pseudogulbenkiania subflava* P19
Brevibacillus schisleri E33	*Massilia phosphatilytica* Y2	*Pseudomonas benzenivorans* P1
Brevibacterium epidermidis R-3-20	*Microbacterium fluvii* Y24	*Pseudomonas cuatrocienegasensis* P94
Burkholderia anthina R25	*Neobacillus drentensis* NewY24	*Pseudomonas furukawaii* P86
Burkholderia anthina R-4183	*Neobacillus drentensis* NewY65	*Pseudomonas oryzae* P24
Burkholderia diffusa R11	*Nocardioides luteus* Y-3-1	*Pseudoxanthomonas indica* P15
Burkholderia diffusa R-15930	*Nocardioides luteus* Y-3-5	*Pseudoxanthomonas mexicana* P-1-2
Burkholderia seminalis R-24196	*Nocardioides luteus* Y-3-8	*Rhodococcus aetherivorans* P-1-43
Burkholderia seminalis R100	*Noviherbaspirillum soli* Y-1-36	*Rhodococcus qingshengii* P-1-48
Burkholderia ubonensis 57	*Noviherbaspirillum* sp. Y-1-9	*Sphingobacterium alimentarium* P-2-35
Burkholderia ubonensis NewR15	*Novosphingobium subterraneum* Y20	*Streptomyces narbonensis* P-2-9
Burkholderiater ritorii R56	*Ochrobactrum intermedium* Y-1-3	*Variovorax soli* P-5-1
Caballeronia peredens R-1-36	*Ochrobactrum pseudintermedium* Y-2-2	

Chryseobacterium cucumeris NewR19	*Paenibacillus polymyxa* NewY35	
Citrobacter portucalensis A60	*Pseudarthrobacter niigatensis* Y-2-36	
Citrobacter portucalensis R83	*Pseudarthrobacter oxydans* Y32	
Comamonas testosteroni E9	*Pseudarthrobacter siccitolerans* Y13	
Comamonas testosteroni R-1-20	*Pseudoduganella eburnea* Y30	
Comamonas testosteroni R-2-33	*Pseudogulbenkiania subflava* Y68	
Cytobacillus firmus E26	*Pseudomonas chlororaphis subsp. aureofaciens* Y72	
Cytobacillus oceanisediminis E47	*Pseudomonas furukawaii* NewY48	
Delftia acidovorans R60	*Pseudomonas furukawaii* NewY60	
Delftia acidovorans R67	*Pseudomonas guariconensis* Y104	
Dyella jiangningensis R-1-34	*Pseudomonas hunanensis* Y115	
Dyella jiangningensis R-2-41	*Pseudomonas nitroreducens* NewY56	
Dyella jiangningensis R-2-45	*Pseudomonas chlororaphis subsp.aureofaciens* Y81	
Falsibacillus pallidus R20	*Pseudomonas guariconensis* Y12	
Falsibacillus pallidus R28	*Pseudomonas hunanensis* Y71	
Fictibacillus halophilus E48	*Rhizobium pusense* Y6	
Kocuria carniphila Hv13-1	*Sphingobium fuliginis* Y33	
Lysinibacillus macroides R-3-9	*Staphylococcus capitis subsp. capitis* Y37	
Lysinibacillus xylanilyticus R-3-1		
Massilia arvi R-1-47		
Massilia terrae J11		
Massilia terrae R23		
Mesorhizobium qingshengii R-2-23		
Mesorhizobium soli R-3-11		
Mesorhizobium soli R-3-36		
Mesorhizobium soli R99		
Mesorhizobium sp. R112		
Methylobacterium oryzae R-3-39		
Microbacterium azadirachtae R88		
Microvirga makkahensis R-1-35		
Paenibacillus cucumis R105		
Paenibacillus cucumis R106		
Paenibacillus silvae R38		
Paenibacillus silvae R91		
Pandoraea fibrosis R50		

Pandoraea fibrosis R53		
Paraburkholderia aromaticivorans R-1-43		
Pseudochrobactrum saccharolyticum E35		
Pseudomonas geniculata E15		
Pseudomonas koreensis NewR37		
Pseudomonas kribbensis NewR13		
Pseudomonas kribbensis NewR34		
Pseudomonas plecoglossicida R6		
Ralstonia syzygii subsp. celebesensis UQRS 627		
Ralstonia syzygii subsp. indonesiensis UQRS 464		
Ralstonia syzygii subsp.celebesensis R5		
Ralstonia syzygii subsp.indonesiensis R43		
Rhizobium radiobacter R62		
Rummeliibacillus stabekisii R108		
Sinomonas atrocyanea R-2-32		
Sinomonas atrocyanea R-2-34		
Sinomonas atrocyanea R-2-44		
Sinomonas atrocyanea R-3-10		
Sinomonas atrocyanea R-3-37		
Sinomonas soli R-1-40		
Sinomonas soli R95		
Sinomonas sp. R-1-42		
Sphingobacterium alimentarium E44		
Sphingobacterium mizutaii E12		
Sphingobacterium olei Sh10		
Sphingopyxis macrogoltabida R104		
Sphingopyxis soli BL03 R104		
Sphingopyxis soli R104		
Sporosarcina luteola R-2-9		
Sporosarcina thermotolerans R-2-7		
Staphylococcus warneri R-2-40		
Staphylococcus warneri R93		
Terrabacter lapilli R-1-41		

Terrabacter lapilli R-1-49		
Terrabacter lapilli R-2-43		
Terrabacter lapilli R-2-46		
Trinickia diaoshuihuensis R21		

Supplementary Table 2. ASV information of keystones and comparison with identity of genomes.

Genus	ASV by random forest analysis	Isolates	Identity[a]	Genome accession[*]	Identity[b]
Comamonas	ASV1	7D-2	100 %	From this project	100 %
Pseudoxanthomonas	ASV2	X-1	100 %	From this project	100 %
Pseudarthrobacter	ASV8	Y13	100 %	GCA_001046895.1	100 %
Sphingobacterium	ASV3	E44	100 %	GCA_004342685.1	100 %
Bacillus	ASV39	P56	100 %	GCA_900101665.1	100 %
Sphingomonas	ASV9	R1	97.67 %	GCA_000016765.1	100 %
Lysinibacillus	ASV305	LM5	97.67 %	GCA_001281525.1	100 %
Pigmentiphaga	ASV1177	H8	100 %	From this project	100 %
Arthrobacter	ASV1564	AT5	98.67 %	From this project	100 %
Aliihoeflea	ASV225	A8	99.67 %	GCA_000497755.1	98.77 %
Sinomonas	ASV2087	R3	99.33 %	GCA_001577305.1	100 %
Bradyrhizobium	ASV157	BR1	100 %	GCA_003062285.1	100 %
Acinetobacter	ASV1311	AC6	94.67 %	GCA_002370525.2	100 %
Nocardioides	ASV130	Y3	100 %	GCA_014648595.1	100 %
Achromobacter	ASV1339	E3	99.33 %	GCA_902860065.1	100 %
Pseudomonas	ASV1886	P1	100 %	GCA_900100495.1	100 %
Streptomyces	ASV1183 (in yellow cinnamon soil)	B2	99.67 %	GCA_000720395.1	100 %
	ASV1013 (in purple soil)	P29	100 %	GCA_014649015.1	99.93 %

[a]Similarity between 16s of the ASV and of respective sequence of selected strains.

[b]Similarity between 16s of the selected strains and of respective sequence in the genome sequence.

[*]Genomes of the mostly closely related species of each strain with available genome sequences were chosen for model construction.

Supplementary Table 3. Strains used in the study and general features of the metabolic models constructed for these strains

Strains	Source	Genes in model	Reactions in model	Metabolites in model	Exchange reactions
Comamonas sp. 7D-2	Chen et al.[48]	878	1585	1596	86
Pseudoxanthomonas sp. X-1	Ruan et al.[51]	711	1511	1524	72
Pigmentiphaga sp. H8	Chen et al.[53]	1002	1670	1665	101
Sphingobacterium sp. E44	Isolated from *in situ* soil	593	1271	1242	60
Bacillus sp. P56	Isolated from *in situ* soil	999	1666	1615	127
Sphingomonas sp. R1	Isolated from *in situ* soil	961	1472	1511	67
Lysinibacillus sp. LM5	Isolated from *in situ* soil	743	1418	1395	83
Streptomyces sp. P29	Isolated from *in situ* soil	988	1575	1599	79
Pseudarthrobacter sp. Y13	Isolated from *in situ* soil	871	1516	1468	88
Arthrobacter sp. AT5	Isolated from *in situ* soil	1337	1680	1598	118
Nocardioides sp. Y3	Isolated from *in situ* soil	824	1513	1514	82
Achromobacter sp. E3	Isolated from *in situ* soil	926	1609	1609	98
Acinetobacter sp. AC6	Isolated from *in situ* soil	748	1453	1452	80
Sinomonas sp. R3	Isolated from *in situ* soil	876	1512	1480	95
Bradyrhizobium sp. BR1	Isolated from *in situ* soil	1140	1664	1691	76
Aliihoeflea sp. A8	Shared strain from Xiamen University	760	1482	1488	68
Streptomyces sp. B2	Bought from Ningbo Mingzhou Biotechnology Co., LTD	945	1574	1584	79
Pseudomonas sp. P1	Isolated from *in situ* soil	964	1665	1639	103

Supplementary Table 4. Half-reactions and their Gibb's standard free energy at PH = 7.0.

Reaction Number	**Half-Reaction**	**$\Delta G^{0'}$** (KJ/e$^-$ eq)
**Combination of X-1 & 7D-2**		
Ra	$0.2500\ O_2 + H^+ + e^- \rightarrow 0.5000\ H_2O$	-79.55
Rd	$0.3043\ CO_2 + 0.0435\ NH_3 + 0.0870\ HBr + H^+ + e^- \rightarrow 0.0435\ C_7H_2Br_2NO + 0.5652\ H_2O$	29.94
Re	$0.0435\ C_7H_2Br_2NO + 0.0652\ H_2O + 0.2500\ O_2 \rightarrow 0.3043\ CO_2 + 0.0435\ NH_3 + 0.0870\ HBr$	-109.49
Rc	$0.2000\ CO_2 + 0.0500\ HCO_3^- + 0.0500\ NH_4^+ + H^+ + e^- \rightarrow 0.0500\ C_5H_7O_2N + 0.4500\ H_2O$	-
Rs	$0.0435\ C_7H_2Br_2NO + 0.1652\ H_2O + 0.0065\ NH_3 \rightarrow 0.0500\ C_5H_7O_2N + 0.0543\ CO_2 + 0.0870\ HBr$	-
R	$0.0435\ C_7H_2Br_2NO + 0.1500\ O_2 + 0.1052\ H_2O \rightarrow 0.0200\ C_5H_7O_2N + 0.2043\ CO_2 + 0.0236\ NH_3 + 0.0870\ HBr$	-
**X-1**		
Ra	$0.1495\ CO_2 + 0.0911\ NH_3 + 0.15\ O_2 \rightarrow 0.0022\ C_5H_9NO_2 + 0.0208\ C_5H_4N_4O + 0.0058\ C_6H_{14}NO_5 + 0.5450\ H_2O$	-30.60
Rd	$0.4362\ CO_2 + 0.2482\ NH_3 + H^+ + e^- \rightarrow 0.0600\ C_5H_4N_4O_2 + 0.0158\ C_6H_{12}O_6 + 0.0082\ C_5H_8NO_4 + 0.6244\ H_2O$	45.97
Re	$0.0600\ C_5H_4N_4O_2 + 0.0158\ C_6H_{12}O_6 + 0.0082\ C_5H_8NO_4 + 0.1500\ O_2 + 0.0794\ H_2O \rightarrow 0.0022\ C_5H_9NO_2 + 0.0208\ C_5H_4N_4O + 0.0058\ C_6H_{14}NO_5 + 0.2867\ CO_2 + 0.1571\ NH_3$	-76.57
Rc	$1/5\ CO_2 + 1/20\ HCO_3^- + 1/20\ NH_4 + H^+ + e^- \rightarrow 1/20\ C_5H_7O_2N + 9/20\ H_2O$	-
Rs	$0.0600\ C_5H_4N_4O_2 + 0.0158\ C_6H_{12}O_6 + 0.0082\ C_5H_8NO_4 + 0.2244\ H_2O \rightarrow 0.0500\ C_5H_7O_2N + 0.1862\ CO_2 + 0.1982\ NH_3$	-
R	$0.0600\ C_5H_4N_4O_2 + 0.0158\ C_6H_{12}O_6 + 0.0082\ C_5H_8NO_4 + 0.0900\ O_2 + 0.1374\ H_2O \rightarrow 0.0200\ C_5H_7O_2N + 0.0013\ C_5H_9NO_2 + 0.0125\ C_5H_4N_4O + 0.0035\ C_6H_{14}NO_5 + 0.2465\ CO_2 + 0.1735\ NH_3$	-
**7D-2**		
Ra	$0.1744\ CO_2 + 0.0992\ NH_3 + 0.1500\ O_2 + H^+ + e^- \rightarrow 0.0240\ C_5H_4N_4O_2 + 0.0632\ C_6H_{12}O_6 + 0.0328\ C_5H_8NO_4 + 0.5498\ H_2O$	-61.70
Rd	$0.3312\ CO_2 + 0.1144\ NH_3 + 0.0538\ HBr + H^+ + e^- \rightarrow 0.0269\ C_7H_2Br_2NO + 0.0020\ C_5H_9NO_2 + 0.0200\ C_5H_4N_4O + 0.0055\ C_6H_{14}NO_5 + 0.5840\ H_2O$	34.88
Re	$0.0269\ C_7H_2Br_2NO + 0.0020\ C_5H_9NO_2 + 0.0200\ C_5H_4N_4O + 0.0055\ C_6H_{14}NO_5 + 0.1500\ O_2 + 0.0344\ H_2O \rightarrow 0.0240\ C_5H_4N_4O_2 + 0.0632\ C_6H_{12}O_6 + 0.0328\ C_5H_8NO_4 + 0.0538\ HBr + 0.1568\ CO_2 + 0.0152\ NH_3$	-96.58
Rc	$1/5CO_2 + 1/20\ HCO_3^- + 1/20\ NH_4 + H^+ + e^- \rightarrow 1/20\ C_5H_7O_2N + 9/20\ H_2O$	-
Rs	$0.0269\ C_7H_2Br_2NO + 0.0020\ C_5H_9NO_2 + 0.0200\ C_5H_4N_4O + 0.0055\ C_6H_{14}NO_5 + 0.1480\ H_2O \rightarrow 0.05\ C_5H_7O_2N + 0.0812\ CO_2 + 0.0644\ NH_3 + 0.0538\ HBr$	-
R	$0.0269\ C_7H_2Br_2NO + 0.0020\ C_5H_9NO_2 + 0.0200\ C_5H_4N_4O + 0.0055\ C_6H_{14}NO_5 + 0.0900\ O_2 + 0.0798\ H_2O \rightarrow 0.0200\ C_5H_7O_2N + 0.0144\ C_5H_4N_4O_2 + 0.0379\ C_6H_{12}O_6 + 0.0197\ C_5H_8NO_4 + 0.0538\ HBr + 0.1266\ CO_2 + 0.0349\ NH_3$	-

Ra, the electron-acceptor equation; *Rd*, the electron-donor equation; *Re*, the energy equation (*Re* = *Ra* - *Rd*); *Rc*, the cell synthesis equation; *Rs*, the synthesis equation (*Rs* = *Rc* - *Rd*); *R*, the overall equation that includes energy generation and synthesis (*R* = *fe* * *Re* + *fs* * *Rs*); *fe*, the portion of electrons used for energy (here, *fe* = 0.6); *fs*, the portion of electrons used for cell synthesis (here, *fs* = 0.4). The free energies of formation for various chemical species ($\Delta_f G$) used in *Ra*, *Rd* and *Re* are obtained from eQuilibrator (https://equilibrator.weizmann.ac.il/)[1]. As no

$\Delta_f G$ for bromoxynil in any public database or literature, we used $\Delta_f G$ for 4-Hydroxybenzoate (the first intermediate metabolite of bromoxynil degradation with available $\Delta_f G$) for calculation. The empirical biomass formula for bacterial cell, $C_5H_7O_2N$, was used[2].

Supplementary Table 5. The overall energy generation and energy requirement for cell synthesis.

	**Δ*Gs***	**Δ*Gr***	**Y/g cells/mol donor**
**X-1&7D-2**	39.92	-109.49	51.95 g cells/(1 mol bromoxynil)
**X-1**	24.81	-76.57	37.60 g cells/(1 mol xanthine + 0.26 mol mannose + 0.14 mol glutamate)
**D-2**	31.68	-96.58	84.01 g cells/(1 mol bromoxynil + 0.07mol proline + 0.74 mol hypoxanthine + 0.20 mol glutamate)

Δ*Gs*, the energy requirement for cell synthesis; Δ*Gr*, the energy released per equivalent of donor oxidization for energy generation; Y, the yield of cells. These results show that the predicted metabolic interaction is thermodynamic feasible ($\Delta Gs + \Delta Gr < 0$).

Supplementary Table 6. Detailed information about the metabolic reactions related to the metabolic interactions between strains X-1 and 7D-2. The standard Gibbs energy of reaction for each reaction ($\Delta_r G'^{o}$) were calculated at pH 7.0, ionic strength of 0.25 M and temperature of 298.15 K by ModelSeed (https://modelseed.org/biochem/reactions) and eQuilibrator (http://equilibrator.weizmann.ac.il).

**Reaction_ID**	**$\Delta_r G'^{o}$ (kcal/mol)**	**EC_ID**	**KEGG_ID**	**Formula**	**Gene_ID**
**X-1**					
rxn01200	0.35	2.2.1.1	R01641	Glyceraldehyde3-phosphate + Sedoheptulose7-phosphate <=> ribose-5-phosphate + D-Xylulose5-phosphate	assembly_01277
rxn00770	0.19	2.7.6.1	R01049	ATP + ribose-5-phosphate => AMP + H+ + PRPP	assembly_03051
rxn00790	-12.98	2.4.2.14	R01072	PPi + L-Glutamate + H+ + 5-Phosphoribosylamine <=> H2O + L-Glutamine + PRPP	assembly_02969
rxn02895	-6.35	6.3.4.13	R04144	ATP + Glycine + 5-Phosphoribosylamine => ADP + Phosphate + H+ + GAR	assembly_00768
rxn03004	-6.68	2.1.2.2	R04325	10-Formyltetrahydrofolate + GAR => H+ + Tetrahydrofolate + N-Formyl-GAR	assembly_01536
rxn03005	-7.29	2.1.2.2	R04326	(2) H+ + Tetrahydrofolate + N-Formyl-GAR <=> H2O + 5-10-Methenyltetrahydrofolate + GAR	assembly_01536
rxn03084	-13.45	6.3.5.3	R04463	H2O + ATP + L-Glutamine + N-Formyl-GAR => ADP + Phosphate + L-Glutamate + H+ + 5'-Phosphoribosylformylglycinamidine	assembly_03367
rxn02937	-20.66	6.3.3.1	R04208	ATP + 5'-Phosphoribosylformylglycinamidine <=> ADP + Phosphate + H+ + AIR	assembly_01537
rxn05114	-13.69	6.3.4.18	R07404	ATP + H2CO3 + AIR => ADP + Phosphate + (2) H+ + 5-phosphoribosyl-5-carboxyaminoimidazole	assembly_02910
rxn05115	-7.71	5.4.99.18	R07405	H+ + 5-phosphoribosyl-5-carboxyaminoimidazole <=> 5'-Phosphoribosyl-4-carboxy-5-aminoimidazole	assembly_02911
rxn03147	-15.5	6.3.2.6	R04591	ATP + L-Aspartate + 5'-Phosphoribosyl-4-carboxy-5-aminoimidazole => ADP + Phosphate + (2) H+ + SAICAR	assembly_00635
rxn03136	3.52	4.3.2.2	R04559	SAICAR <=> Fumarate + AICAR	assembly_01833
rxn03137	2.56	2.1.2.3	R04560	10-Formyltetrahydrofolate + AICAR => Tetrahydrofolate + FAICAR	assembly_00769
rxn00832	-4.15	2.1.2.3	R01127	FAICAR <=> H2O + IMP	assembly_00769
rxn00834	4.75	1.1.1.205	R01130	H2O + NAD + IMP <=> NADH + H+ + XMP	assembly_02033
rxn01961	-2.65	3.1.3.5	R02719	H2O + XMP <=> Phosphate + Xanthosine	assembly_01688
rxn01547	8.88	3.5.4.15	R02145	NH3 + Xanthosine <=> H2O + H+ + Guanosine	assembly_01064
rxn01226	-0.45	3.2.2.1	R01677	H2O + Guanosine <=> D-Ribose + Guanine	assembly_02904
rxn01225	-8.88	3.5.4.3	R01676	H2O + H+ + Guanine => NH3 + XAN	assembly_01945
rxn01297	-3.8	1.17.1.4	R01768	NADH + H+ + XAN <=> H2O + NAD + HYXN	assembly_02298

rxn00931	-4.72	1.5.1.2	R01251	NADPH + (2) H+ + 1-Pyrroline-5-carboxylate <=> NADP + L-Proline	assembly_01106
rxn00503	2.27	1.5.1.12	R00707	NADH + L-Glutamate + H+ <=> (2) H2O + NAD + 1-Pyrroline-5-carboxylate	assembly_03695
rxn00085	11.53	1.4.1.13	R00114	NADP + (2) L-Glutamate <=> NADPH + 2-Oxoglutarate + L-Glutamine + H+	assembly_00038
rxn00555	-3.99	2.6.1.16	R00768	L-Glutamine + D-fructose-6-phosphate <=> L-Glutamate + D-Glucosamine phosphate	assembly_00320
rxn05569	8.57	-	-	Pyruvate + D-Glucosamine phosphate <=> Phosphoenolpyruvate + GLUM[1]	assembly_01975
rxn50000	-	3.1.1.1	-	BO => Bromoxynil + octanoate	assembly_00263
rxn05610	-9.58	-	-	Phosphoenolpyruvate + D-Mannose[1] <=> Pyruvate + D-mannose-6-phosphate	assembly_01975
rxn00559	-0.21	5.3.1.8	R00772	D-mannose-6-phosphate <=> D-fructose-6-phosphate	assembly_01543
**7D-2**					
rxn00182	-8.71	1.4.1.2	R00243	NADH + NH3 + 2-Oxoglutarate + H+ <=> H2O + NAD + L-Glutamate	7D_2_CD1.Chr000122
rxn00199	-3.33	1.1.1.42	R00268	H+ + Oxalosuccinate => CO2 + 2-Oxoglutarate	7D_2_CD1.Chr003020
rxn01387	4.75	1.1.1.42	R01899	NADP + Isocitrate <=> NADPH + H+ + Oxalosuccinate	7D_2_CD1.Chr003020
rxn00973	1.82	4.2.1.3	R01324	Citrate <=> Isocitrate	7D_2_CD1.Chr003547
rxn00256	8.6	2.3.3.1	R00351	H2O + Acetyl-CoA + Oxaloacetate => CoA + H+ + Citrate	7D_2_CD1.Chr001323
rxn00258	0.02	2.1.3.1	R00353	Pyruvate + Malonyl-CoA <=> Acetyl-CoA + Oxaloacetate	7D_2_CD1.Chr002711
rxn00004	4.49	4.1.3.17	R00008	Parapyruvate <=> (2) Pyruvate	7D_2_CD1.Chr002953
rxn03442	-0.38	4.2.1.-	R05078	Parapyruvate <=> H2O + 4-Methylene-2-oxoglutarate	-
rxn11610	-9.31	3.7.1.-	R04488	H2O + 4-Carboxy-2-hydroxymuconate semialdehyde => Formate + H+ + 4-Methylene-2-oxoglutarate	-
rxn01193	-61.57	1.13.11.8	R01632	O2 + Protocatechuate => H+ + 4-Carboxy-2-hydroxymuconate semialdehyde	7D_2_CD1.Plasmid000020
rxn00962	-105.96	1.14.13.2	R01298	NADPH + O2 + H+ + 4-Hydroxybenzoate => H2O + NADP + Protocatechuate	7D_2_CD1.Plasmid000020
rxn47165	-	-	-	(2) H2O + NADP + 3-bromo-4-hydroxybenzoate <=> NADPH + O2 + (2) H+ + 4-Hydroxybenzoate + HBr	7D_2_CD1.Plasmid000019
rxn50001	-	-	-	H2O + 3,5-Dibromo-4-hydroxybenzoate => 3-bromo-4-hydroxybenzoate + HBr	7D_2_CD1.Plasmid000019
rxn03021	0.26	3.5.5.6	R04349	(2) H2O + Bromoxynil => NH3 + 3,5-Dibromo-4-hydroxybenzoate	7D_2_CD1.Plasmid000030
rxn00148	6.53	2.7.1.40	R00200	ATP + Pyruvate <=> ADP + Phosphoenolpyruvate + H+	7D_2_CD1.Chr00

					4869
rxn00459	0.98	4.2.1.11	R00658	H2O + Phosphoenolpyruvate <=> 2-Phospho-D-glycerate	7D_2_CD1.Chr001178
rxn01106	-1	5.4.2.1	R01518	2-Phospho-D-glycerate <=> 3-Phosphoglycerate	7D_2_CD1.Chr000889
rxn01100	4.42	2.7.2.3	R01512	ATP + 3-Phosphoglycerate <=> ADP + 1,3-Bisphospho-D-glycerate	7D_2_CD1.Chr000418
rxn00781	-1.25	1.2.1.12	R01061	NADH + H+ + 1,3-Bisphospho-D-glycerate <=> NAD + Phosphate + Glyceraldehyde3-phosphate	7D_2_CD1.Chr004881
rxn00613	-7.45	1.1.1.177	R00845	NADPH + H+ + Glyceraldehyde3-phosphate <=> NADP + Glycerol-3-phosphate	7D_2_CD1.Chr004068
rxn00786	-5.12	4.1.2.13	R01068	Glycerone-phosphate + Glyceraldehyde3-phosphate <=> D-fructose-1,6-bisphosphate	7D_2_CD1.Chr004865
rxn00545	4.64	2.7.1.11	R00756	ADP + H+ + D-fructose-1,6-bisphosphate <=> ATP + D-fructose-6-phosphate	7D_2_CD1.Chr001040
rxn00559	0.21	5.3.1.8	R00772	D-fructose-6-phosphate <=> D-mannose-6-phosphate	7D_2_CD1.Chr004443
rxn00975	3.05	2.7.1.1	R01326	ADP + H+ + D-mannose-6-phosphate <=> ATP + D-Mannose	7D_2_CD1.Chr000321
rxn01297	3.8	1.17.1.4	R01768	H2O + NAD + HYXN <=> NADH + H+ + XAN	7D_2_CD1.Chr000321

Supplementary Table 7. LC-MS analysis of the metabolites in consortium samples of BO groups. BO, bromoxynil octanoate; BRO, bromoxynil. X-1&7D-2&BR1&BO, the three strains were inoculated at the same time. (7D-2&BR1&BRO-4h)+X-1, the strains 7D-2 and BR1 were cultured in the MM medium containing BRO for 4 hours, and then the strain X-1 was added. Excretion of (7D-2&BR1&BRO-4h)+X-1, the strains 7D-2 and BR1 were cultured in the MM medium containing BRO for 4 hours and centrifuged to remove the bacteria, and then the strain X-1 was added. 7D-2&BR1&BRO, the strains 7D-2 and BR1 were inoculated at the same time. All final cultures were tested after 4 hours of culture.

	X-1&7D-2&BR1&BO	(7D-2&BR1&BRO-4h)+X-1	Excretion of (7D-2&BR1&BRO-4h)+X-1	BR1&BRO	7D-2&BR1&BRO
Hypoxanthine	+	+	+	-	-
L-Glutamate	+	+	+	-	-
Xanthine	+	+	+	-	-
Fumarate	+	+	+	-	+
4-Hydroxybenzoate	+	+	+	-	+
Succinate	+	+	+	-	+
L-Lysine	+	+	-	-	+
(R)-3-Hydroxybutanoate	+	+	+	-	+

Supplementary Table 8. Information of reactions adding to the synthetic cell.

Reaction ID	ER/HR*	Origin	Equation
rxn50000	ER	Strain X-1	Bromoxynil octanoate => Bromoxynil + Octanoate
rxn03021	ER	Strain 7D-2	(2) H_2O + Bromoxynil => NH_4^+ + 3,5-Dibromo-4-hydroxybenzoate
rxn50001	ER	Strain 7D-2	3,5-Dibromo-4-hydroxybenzoate + H^+ =>3-Bromo-4-hydroxybenzoate + HBr
rxn47165	ER	Strain 7D-2	NADPH + O_2 + (2) H^+ + 4-Hydroxybenzoate + HBr <=> (2) H_2O + NADP + 3-Bromo-4-hydroxybenzoate
rxn00960	ER	Strain 7D-2	NADH + O_2 + H^+ + 4-Hydroxybenzoate => H_2O + NAD + Protocatechuate
rxn00962	ER	Strain 7D-2	NADPH + O_2 + H^+ + 4-Hydroxybenzoate => H_2O + NADP + Protocatechuate
rxn01193	ER	Strain 7D-2	O_2 + Protocatechuate => H^+ + 4-Carboxy-2-hydroxymuconate semialdehyde
rxn11610	ER	Strain 7D-2	H_2O + 4-Carboxy-2-hydroxymuconate semialdehyde => Formate + H^+ + 4-Methylene-2-oxoglutarate
rxn03442	ER	Strain 7D-2	Parapyruvate <=> H_2O + 4-Methylene-2-oxoglutarate
rxn00004	ER	Strain 7D-2	Parapyruvate <=> (2) Pyruvate
rxn07597	ER	Strain H8	3,5-Dibromo-4-hydroxybenzoate <=> 2,6-Dibromophenol
rxn07598	ER	Strain H8	2,6-Dibromophenol <=> 2,6-Dibromohydroquinone
rxn07599	ER	Strain H8	2,6-Dibromohydroquinone <=> 2-Bromomaleylacetate
rxn07595	ER	Strain H8	NADH + 2-Bromomaleylacetate <=> NAD + HBr + Maleylacetate
rxn02141	ER	Strain H8	NAD + 3-Oxoadipate <=> NADH + H^+ + Maleylacetate
rxn02142	ER	Strain H8	NADP + 3-Oxoadipate <= NADPH + H^+ + Maleylacetate
rxn02143	ER	Strain H8	Succinyl-CoA + 3-Oxoadipate <= Succinate + 3-Oxoadipyl-CoA
rxn00598	ER	Strain H8	Acetyl-CoA + Succinyl-CoA <= CoA + 3-Oxoadipyl-CoA
rxn10120	ER	Strain BR1	H^+ + Nitrate + Ubiquinol-8 => H_2O + Nitrite + Ubiquinone-8
rxn10121	ER	Strain BR1	H^+ + Nitrate + Menaquinol 8 => H_2O + Nitrite + Menaquinone 8
rxn05893	ER	Strain BR1	(2) H_2O + NH_4^+ + (6) Oxidizedferredoxin <= (7) H^+ + Nitrite + (6) Reducedferredoxin
rxn00274	HR	Strain X-1	Acetyl-CoA + Glycine <=> CoA + L-2-Amino-acetoacetate
rxn06556	HR	Strain X-1, BR1	H_2O + Dodecanoyl-ACP => H^+ + Dodecanoic acid + ACP
rxn09452	HR	Strain X-1	ATP + CoA + Dodecanoic acid => PPi + AMP + H+ + Lauroyl-CoA

*ER, essential reaction; HR, helpful reaction.

Supplementary Table 9. Detailed information about standard metabolites detected by LC-MS.

**Metabolites**	**Mass**	**Retention time (min)**	**Positive/negative ion mode**
D-Glucosamine	180.0872	0.79	+
L-Lysine	147.1134	0.75	+
L-Proline	116.0712	0.90	+
Xanthine	153.0413	1.29	+
L-Glutamate	147.0610	1.52	+
Hypoxanthine	137.0463	1.19	+
(R)-3-Hydroxybutanoate	103.0395	1.67	-
Fumarate	115.0031	1.54	-
D-mannose	179.0566	0.85	-
Succinate	117.0188	1.57	-

Supplementary References

1. Beber, M. E. et al. eQuilibrator 3.0: a database solution for thermodynamic constant estimation. *Nucleic Acids Res.* **50**, D603-D609 (2022).
2. Rittmann, B. E. & McCarty, P. L. Environmental Biotechnology: Principles and Applications, Second Edition. (New York, N.Y.: McGraw-Hill Education, 2020).
