# Supplementary material for: Engineering natural microbiomes toward enhanced bioremediation by microbiome modeling
Source: Nat Commun. 2024 Jun 1;15:4694. doi: 10.1038/s41467-024-49098-z (PMC11144243; doi:10.1038/s41467-024-49098-z)
Supplement: Supplementary file 3 — Description of Additional Supplementary Files [file 41467_2024_49098_MOESM3_ESM.pdf]

Description of Additional Supplementary Files:

Supplementary Data 1: Genome-scale metabolic models of the 18 strains used in this study.
