# Supplementary material for: Engineering natural microbiomes toward enhanced bioremediation by microbiome modeling
Source: Nat Commun. 2024 Jun 1;15:4694. doi: 10.1038/s41467-024-49098-z (PMC11144243; doi:10.1038/s41467-024-49098-z)
Supplement: Supplementary file 5 — Reporting Summary [file 41467_2024_49098_MOESM5_ESM.pdf]

nature portfolio

Corresponding author(s): Yanzheng Gao, Jiandong Jiang, Xihui Xu

Last updated by author(s): May 13, 2024

Reporting Summary

Nature Portfolio wishes to improve the reproducibility of the work that we publish. This form provides structure for consistency and transparency in reporting. For further information on Nature Portfolio policies, see our Editorial Policies and the Editorial Policy Checklist.

Statistics

For all statistical analyses, confirm that the following items are present in the figure legend, table legend, main text, or Methods section.

n/a	Confirmed	
☐	☒	The exact sample size (*n*) for each experimental group/condition, given as a discrete number and unit of measurement
☐	☒	A statement on whether measurements were taken from distinct samples or whether the same sample was measured repeatedly
☐	☒	The statistical test(s) used AND whether they are one- or two-sided<br>*Only common tests should be described solely by name; describe more complex techniques in the Methods section.*
☐	☒	A description of all covariates tested
☐	☒	A description of any assumptions or corrections, such as tests of normality and adjustment for multiple comparisons
☐	☒	A full description of the statistical parameters including central tendency (e.g. means) or other basic estimates (e.g. regression coefficient) AND variation (e.g. standard deviation) or associated estimates of uncertainty (e.g. confidence intervals)
☐	☒	For null hypothesis testing, the test statistic (e.g. *F*, *t*, *r*) with confidence intervals, effect sizes, degrees of freedom and *P* value noted<br>*Give P values as exact values whenever suitable.*
☒	☐	For Bayesian analysis, information on the choice of priors and Markov chain Monte Carlo settings
☒	☐	For hierarchical and complex designs, identification of the appropriate level for tests and full reporting of outcomes
☒	☐	Estimates of effect sizes (e.g. Cohen's *d*, Pearson's *r*), indicating how they were calculated

Our web collection on statistics for biologists contains articles on many of the points above.

Software and code

Policy information about availability of computer code

Data collection	Illumina Miseq PE250 platform was used for 16S rRNA gene amplicon sequencing; Illumina NovaSeq 6000 platform was used for metagenomic and transcriptomic sequencing. All statistical analysis were conducted in R (v4.0.3), MATLAB (R2019a), and GraphPad Prism (v8.0).
Data analysis	QIIME (v2); MEGAHIT (v1.1.2); CD-HIT (v4.6.1); SOAPaligner (v2.21); Diamond (v0.8.35); Trimmomatic (v0.36); Unicycler (v0.4.8); tRNAscan-SE (v1.23); RNAmmer (v1.2); Mothur (v1.21.1); Vegan (v2.5-7), randomForest (v4.6-14), ggplot2 (v3.3.0), pheatmap (v1.0.12) and VennDiagram (v1.6.20) packages in R (v4.0.3). The models were analyzed using COBRAToolbox-3.0 in MATLAB (R2019a). The code used for the analysis is available on GitHub (https://github.com/ruanzhepu/superCC).

For manuscripts utilizing custom algorithms or software that are central to the research but not yet described in published literature, software must be made available to editors and reviewers. We strongly encourage code deposition in a community repository (e.g. GitHub). See the Nature Portfolio guidelines for submitting code & software for further information.

Data

Policy information about availability of data

All manuscripts must include a data availability statement. This statement should provide the following information, where applicable:

- Accession codes, unique identifiers, or web links for publicly available datasets
- A description of any restrictions on data availability
- For clinical datasets or third party data, please ensure that the statement adheres to our policy

There is no restriction on data availability. All amplicon sequencing data have been deposited in the NCBI Sequence Read Archive (SRA) under the accession number

PRJNA799911. The metagenomic sequence data have been deposited in the NCBI SRA under the accession number PRJNA799881. The RNA-sequencing data have been deposited in the NCBI SRA under the accession number PRJNA1073009. The GenBank accession numbers for genome sequences of Pseudoxanthomonas sp. X-1, Pigmentiphaga sp. H8, Arthrobacter sp. AT5, and Comamonas sp. 7D-2 are VCHZ00000000, CP033966, CP136441, and CP094238, respectively. The GenBank accession number for plasmid of Comamonas sp. 7D-2 is KC771559. The data that support this study are available within the article and its Supplementary Information files. Source data are provided with this paper.

Research involving human participants, their data, or biological material

Policy information about studies with human participants or human data. See also policy information about sex, gender (identity/presentation), and sexual orientation and race, ethnicity and racism.

Reporting on sex and gender	Not applicable. The study does not involve any human participants or human data.
Reporting on race, ethnicity, or other socially relevant groupings	Not applicable. The study does not involve any human participants or human data.
Population characteristics	Not applicable. The study does not involve any human participants or human data.
Recruitment	Not applicable. The study does not involve any human participants or human data.
Ethics oversight	Not applicable. The study does not involve any human participants or human data.

Note that full information on the approval of the study protocol must also be provided in the manuscript.

Field-specific reporting

Please select the one below that is the best fit for your research. If you are not sure, read the appropriate sections before making your selection.

[x] Life sciences [ ] Behavioural & social sciences [ ] Ecological, evolutionary & environmental sciences

For a reference copy of the document with all sections, see nature.com/documents/nr-reporting-summary-flat.pdf

Life sciences study design

All studies must disclose on these points even when the disclosure is negative.

Sample size	No sample size calculation was performed. Minimum of 3 biological triplicates were performed.
Data exclusions	A total of 348 soil samples were analyzed by 16S rRNA gene amplicon sequencing. A total of 28 soil samples were found to be contaminated or with low sequencing quality, and were excluded from the analysis.
Replication	Three or four independent experiments were performed under the same conditions to obtain n=3 or n=4. All experiments performed in this study were successful.
Randomization	Sample allocation was random in our study.
Blinding	The investigators were blinded to group allocation during data collection and analysis in this study.

Reporting for specific materials, systems and methods

We require information from authors about some types of materials, experimental systems and methods used in many studies. Here, indicate whether each material, system or method listed is relevant to your study. If you are not sure if a list item applies to your research, read the appropriate section before selecting a response.

Materials & experimental systems

n/a	Involved in the study	
[x]	[ ]	Antibodies
[x]	[ ]	Eukaryotic cell lines
[x]	[ ]	Palaeontology and archaeology
[x]	[ ]	Animals and other organisms
[x]	[ ]	Clinical data
[x]	[ ]	Dual use research of concern
[x]	[ ]	Plants

Methods

n/a	Involved in the study	
[x]	[ ]	ChIP-seq
[x]	[ ]	Flow cytometry
[x]	[ ]	MRI-based neuroimaging

Plants

Seed stocks	*Report on the source of all seed stocks or other plant material used. If applicable, state the seed stock centre and catalogue number. If plant specimens were collected from the field, describe the collection location, date and sampling procedures.*
Novel plant genotypes	*Describe the methods by which all novel plant genotypes were produced. This includes those generated by transgenic approaches, gene editing, chemical/radiation-based mutagenesis and hybridization. For transgenic lines, describe the transformation method, the number of independent lines analyzed and the generation upon which experiments were performed. For gene-edited lines, describe the editor used, the endogenous sequence targeted for editing, the targeting guide RNA sequence (if applicable) and how the editor was applied.*
Authentication	*Describe any authentication procedures for each seed stock used or novel genotype generated. Describe any experiments used to assess the effect of a mutation and, where applicable, how potential secondary effects (e.g. second site T-DNA insertions, mosiacism, off-target gene editing) were examined.*
